# Through the holes: the biotechnological potential of actinoporins (and other PFPs)

**DOI:** 10.1007/s12551-026-01423-0

**Published:** 2026-02-28

**Authors:** Javier Maraver-de-Paz, Diego Heras-Márquez, Juan Palacios-Ortega, Álvaro Martínez-del-Pozo, Sara García-Linares

**Affiliations:** 1 Departamento de Bioquímica y Biología Molecular, Facultad de Ciencias Químicas, Universidad Complutense, Madrid, 28020 Spain; 2 Departamento de Ciencias Médicas Básicas, Facultad de Medicina, Universidad San Pablo-CEU, CEU Universities, Boadilla del Monte, Spain; 3 Department of Chemistry, Faculty of Natural, Mathematical and Engineering Sciences, King’s College London, 7 Trinity Street, SE1 1DB London, UK

**Keywords:** Pore-forming proteins, Nanopores, Protein engineering, Sphingomyelin, Biosensors, Nanoreactors, PET degradation, Microplastic recycling

## Abstract

Pore-forming proteins (PFPs) are singular polypeptides used by all kinds of organisms for attack or defence. They defy the stereotypical classification between water-soluble and membrane proteins. Within this large family of proteins, bacterial hemolysins and sea anemone actinoporins stand out as candidates for transforming these toxins into therapeutic or biotechnological devices. Over the past two decades, many examples have been published where these toxic proteins have been adapted to perform single-molecule tasks such as biosensing, sequencing of proteins and nucleic acids, discriminating protein chemical modifications, proteomics analyses, or even the use of DNA in computational approaches. Lately, PFPs have also been incorporated as templates for designing new artificial nanoreactors to catalyse different chemical reactions. A promising alternative within this idea is the recent publication of a proof of concept showing that actinoporins can be converted into biosustainable plastic-degrading nanoreactors. It is also discussed how optimisation and development of future PFP-based nanoreactors with improved activity and new specificities seem to be a venue worth to explore in order to degrade the contaminating waste material made of plastics from very different chemical compositions.

## Pore-forming-proteins

Pore-forming proteins (PFPs) conform a large, ubiquitous, and fascinating family of proteins found in virtually all kingdoms of life (Chatterjee et al. 2025a; Mori et al. 2024). They have the unique property of being synthesised as stable water-soluble monomers with the ability to bind suitable lipid membranes. Attachment to the membrane increases the local concentration of the protein, reducing diffusion to a bidimensional system and facilitating the oligomerisation leading to pore formation. During this process, they undergo a conformational metamorphosis that allows them to become integral membrane proteins (García-Linares et al. 2017; Iacovache et al. 2010; Kulma and Anderluh 2021; Lella and Mahalakshmi 2017; Margheritis et al. 2023; Tanaka et al. 2015a; Ulhuq and Mariano 2022). Pore size and permeability selectivity vary for different PFPs, allowing the passage of species ranging from small ions to medium-size proteins. Consequently, attacked cells typically die from an osmotic shock, with some examples of intracellular deleterious effects provoked by the action of enzymes penetrating the cytosol via the perforating pores (Ding et al. 2016; Korn and Pluhackova 2025; Wright et al. 2025).

**Fig. 1:**
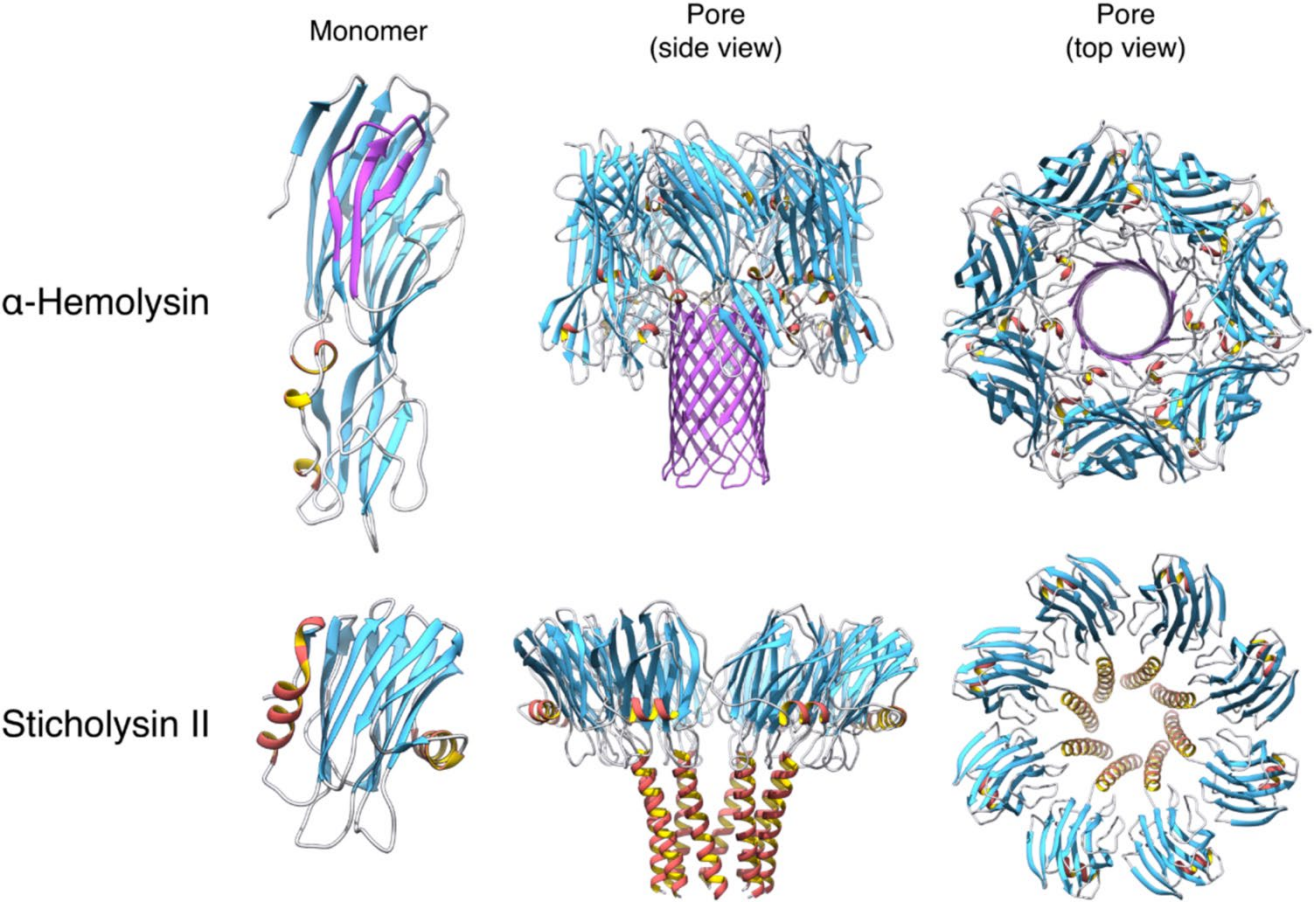
Water-soluble monomeric and pore structures of α-hemolysin from *Staphylococcus aureus* and sticholysin II from the sea anemone *Stichodactyla helianthus*, as representative examples of β- or α-PFPs, respectively. For α-hemolysin, the part that penetrates the membrane has been coloured in purple. PDB IDs: 1GWY (StnII monomer), 9GJ8 (StnII pore), 4YHD (α-hemolysin monomer), and 7AHL (α-hemolysin pore). Graphs and images were built using UCSF Chimera (Pettersen et al. 2004)

PFPs are the main toxins whose activity relies on the disruption of a lipid membrane. They elude the standard dichotomic classification of water-soluble or membrane proteins. Based on the folding of the PFP in its final pore conformation, these proteins are classified either as α-PFPs, if the pore walls are defined by α-helices, or β-PFPs, if the pore walls are β-strands (Arranz et al. 2025; García-Ortega et al. 2011; Johnstone et al. 2025; Margheritis et al. 2023; Parker and Feil 2005) (Fig. 1). According to some authors in the field (Mondal et al. 2022), β-PFPs can be further classified into two subcategories: small β-PFTs, such as *Staphylococcus aureus* α-hemolysin or *Vibrio cholerae* cytolysin (Chatterjee et al. 2025b; Cyr 2018), which generate pores of ~ 1–2 nm diameter, and large β-PFTs, such as perfringolysin O (PFO) or pneumolysin (PLY), that produce β-barrel pores of ~ 30–50 nm diameter, consisting of many more subunits (Johnstone et al. 2025; Liu et al. 2025a; Xia et al. 2021).

PFPs attack the barrier that delimits a cell, the plasma membrane. Hence, most PFPs are toxins, involved in different mechanisms of attack or defence, with a variety of examples including many bacterial toxins (González et al. 2008; Ulhuq and Mariano 2022), or perforin-1, produced by natural killer cells and cytotoxic T lymphocytes (Hodel et al. 2025; Surm et al. 2024). For some PFPs, a protein receptor in the membrane is required to trigger binding and subsequent oligomerisation, as is the case of latrotoxins, highly specialised PFPs from the venom of black widow spiders (Rivera-de-Torre et al. 2019). In many other cases, the receptor is a lipid, as is the case of cholesterol-dependent cytolysins (CDCs) (Evans et al. 2020; Johnstone et al. 2022, 2025; Lata et al. 2024; Ruan et al. 2016; Yu et al. 2022) or actinoporins (Arranz et al. 2025; García-Linares et al. 2016a, 2017; García-Ortega et al. 2011; Palacios-Ortega et al. 2021a), which recognise sphingomyelin (SM). Although PFPs are usually detrimental for human health (at different levels of severity, of course), nowadays researchers succeed in repurposing some of these toxic proteins as therapeutics and witty biotechnological devices (Gupta et al. 2024).

## Actinoporins

**Fig. 2:**
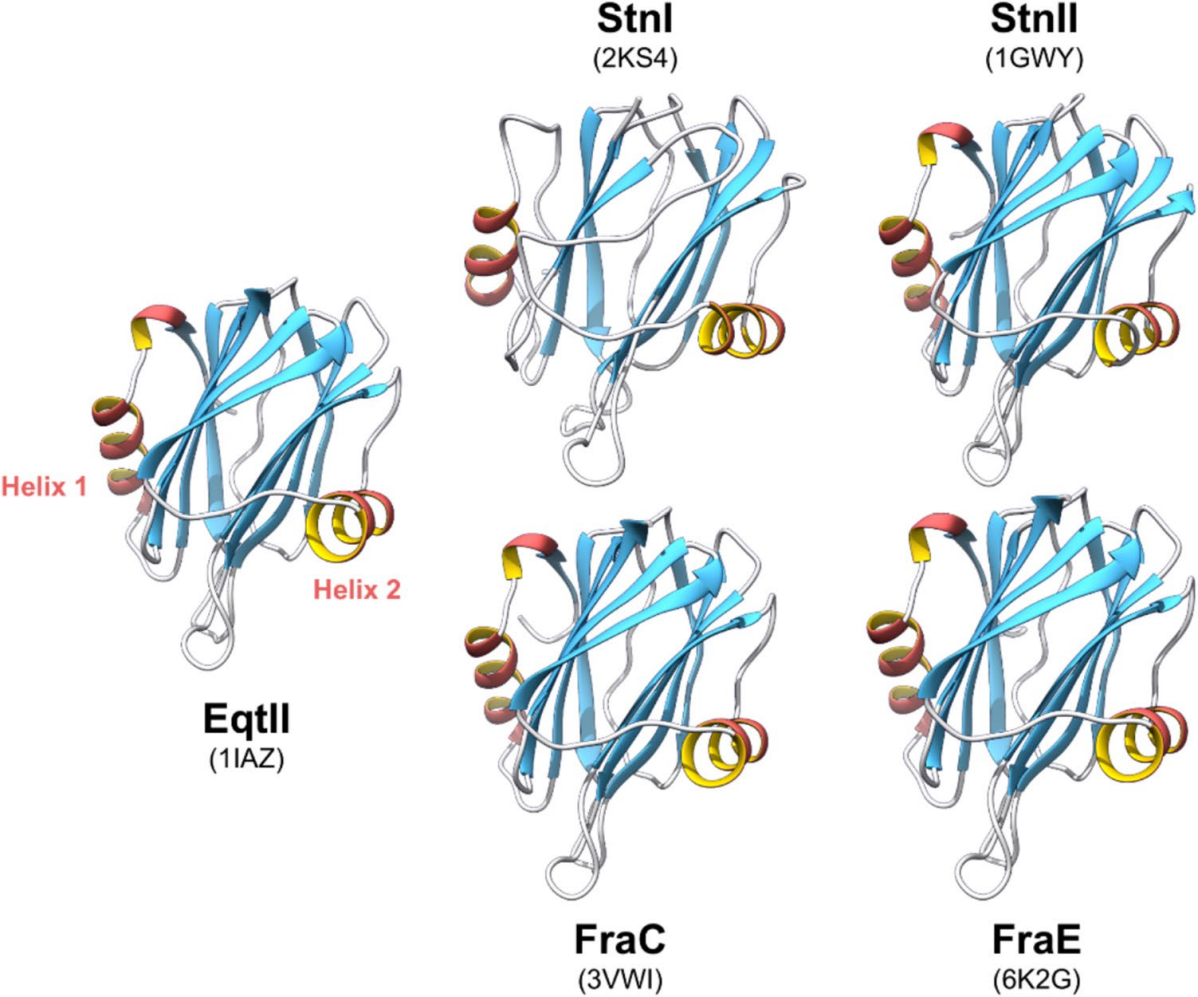
The five resolved three-dimensional structures of actinoporins. Notice the common fold and how, in all structures, both helices lie on the depressions defined by the warp of the β-sheets. Graphs and images were built using UCSF Chimera (Pettersen et al. 2004)

Actinoporins are small-sized and cysteineless proteins (Barroso et al. 2025), secreted by different sea anemones (Alegre-Cebollada et al. 2007; Anderluh et al. 2004; García-Linares et al. 2017; García-Ortega et al. 2011; Maček 1992; Maček et al. 1994; Palacios-Ortega et al. 2021b). They usually display basic pI values and form cation-selective pores (Varanda and Finkelstein 1980). The absence of Cys is greatly advantageous, allowing for the specific introduction of this amino acid for labelling at key locations (Anderluh et al. 1998, 1999a). Actinoporins produce funnel-shaped pores (Fig. 1) of 1–2 nm in diameter at its narrower *trans* side (i.e. cytoplasmic) on their target cells (Arranz et al. 2025; Mancheño et al. 2003; Tanaka et al. 2015b; Tejuca et al. 2001). Generally, actinoporins induce cell death by osmotic shock (De los Ríos et al. 1998; Tejuca et al. 1996). As many other toxins, actinoporins constitute multigene families. One individual can produce different actinoporin isoforms, which are structurally similar but, sometimes, display quite distinct pore-forming activity (Rivera-de-Torre et al. 2016, 2017, 2020). Despite the great number of natural variants of actinoporins described so far (Anderluh et al. 1999b; Leychenko et al. 2018; Valle et al. 2015, 2016; Wang et al. 2008), only the soluble structure of five of them has been resolved in atomic detail (Athanasiadis et al. 2001; García-Linares et al. 2013; Hinds et al. 2002; Mancheño et al. 2003; Mechaly et al. 2009, 2011; Morante et al. 2019; Tanaka et al. 2015b). Sticholysins I and II (StnI and StnII) are both produced by the same Caribbean Sea anemone, *Stichodactyla helianthus* (Martínez et al. 2001), while fragacea toxins (FraC and FraE) are synthesised by the Atlantic Sea anemone *Actinia fragacea* (Bellomio et al. 2009). Finally, equinatoxin II (EqtII) is produced by *Actinia equina* (Anderluh et al. 1997), a commonly occurring sea anemone from the European and Mediterranean coasts. The monomeric water-soluble structures of these five proteins consist of a β-sandwich flanked by two α-helices (Fig. 2). The α-helices rest on the exterior surface of each of the two β-sheets, accommodated in the shallow valleys created by the β-sheet’s warp.

**Fig. 3:**
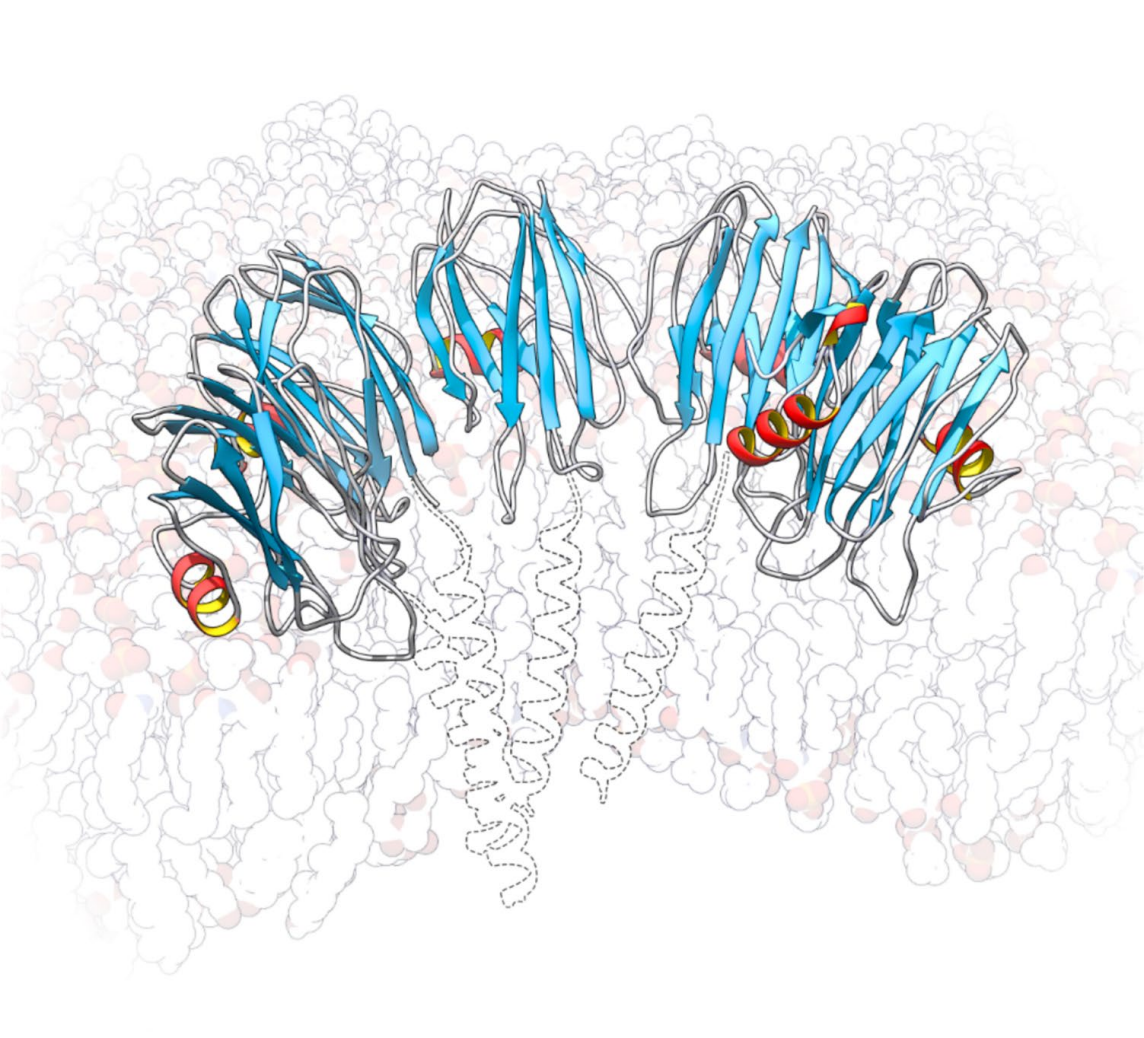
Pore intermediate of StnII. Intermediates of five (shown; PDB ID: 9GKI) and six subunits have been detected and their structure determined (Arranz et al. 2025). Notice that α-helix 1 of the last monomer on the right is still attached to the β-sandwich. The expected positions of the extended N-terminal α-helices of the first four monomers, which could not be determined in these models, are shown as broken lines (placed by aligning PDB 9GJ8 to the intermediate shown). Graphs and images were built using UCSF Chimera (Pettersen et al. 2004)

Actinoporins specifically bind to their target membranes by recognising SM, often considered their receptor (Alegre-Cebollada et al. 2007; Anderluh and Maček 2002; Barlič et al. 2004; Caaveiro et al. 2001; Maček 1992; Schön et al. 2008). The presence of SM is the essential requirement for binding, but several other physicochemical features of the membrane can greatly influence or modulate actinoporin functionality (Alm et al. 2015; Bakrač et al. 2010; Barlič et al. 2004; De los Ríos et al. 1998; García-Linares et al. 2016b; Martínez et al. 2007; Palacios-Ortega et al. 2016; Pedrera et al. 2014; Varanda and Finkelstein 1980). The presence and effect of cholesterol (Chol) in the membrane is possibly the most remarkable of these characteristics (Alm et al. 2015; Arranz et al. 2025; García-Linares et al. 2016a, 2016b; Marchioretto et al. 2013; Martínez et al. 2007; Palacios-Ortega et al. 2016, 2019; Pedrera et al. 2015; Schön et al. 2008; Wacklin et al. 2016), given the abundance of this lipid (or other similar sterols) in eukaryotic membranes. The oligomerisation leading to the cation-selective pores made by actinoporins seems to occur preferentially once the protomers are bound to the membrane (Varanda and Finkelstein 1980). The steps and the stoichiometry of the final thermodynamically stable pore complex seem to be finally solved (Arranz et al. 2025; Mancheño et al. 2003; Martín-Benito et al. 2000; Mechaly et al. 2009; Tanaka et al. 2015b), but the underlying mechanism remains to be explained in full molecular detail. Interestingly, recent cryo-EM studies have shed new light, useful to solve this conundrum (Arranz et al. 2025). Detection of arc-shaped assemblies, corresponding to pore-forming intermediates, allowed to propose the most detailed mechanism to date. Nevertheless, some of its molecular details still remain to be proven. Extension of α-helix 1 as an initial step in pore formation had already been described for EqtII (Rojko et al. 2013) and it is accepted as a key step of the process. Our recent findings corroborate the existence of a sequential mechanism. Monomers seem to be incorporated one by one to an initial monomer in the membrane, causing the arc to grow concomitantly with the helix extension process, until the arc eventually closes into a complete pore (Arranz et al. 2025) (Fig. 3). The monomer at one end of the intermediates retains a structure very similar to the water-soluble form of the protein. Meanwhile, the remaining subunits have α-helix 1 deployed over the membrane. Based on these data, we proposed a mechanism in which a membrane-bound monomer serves as a nucleation point for other monomers to bind by lateral diffusion, leading to pore formation. The mechanism that drives the formation of the pores would be then the result of the lateral monomer-monomer interactions combined with steric clashes among them. These would result in the extension of α-helix 1 on the membrane surface. However, the precise timing of the membrane perforating step remains unclear. It might only occur after the pre-pore arc is closed (Arranz et al. 2025; Morante et al. 2016), but other studies indicate that this would occur before the structure is complete (Cosentino et al. 2016).

## Actinoporins as lipid biosensors

Actinoporin’s recognition of SM is very specific. Taking advantage of this feature, some of them have been engineered to be used as biosensors of this sphingolipid at different cellular and subcellular locations (Makino et al. 2015; Mori et al. 2024; Skocaj et al. 2012; Yachi et al. 2012). Cholera toxin subunit B, which interacts specifically with raft-residing ganglioside GM1, has long been the most commonly used lipid probe for membrane rafts detection. Similarly, given the presence of SM in rafts, a fluorescently labelled EqtII mutant was also used to detect the presence of these so-called detergent resistant domains at the cellular membrane. Besides EqtII, the same work described the rather successful employment of four more toxic proteins with the potential to be used as selective raft markers (Skocaj et al. 2012). Later, EqtII, in conjunction with the CDC toxin lysenin, was also tested as a probe to study the subcellular location of SM in COS-1 cells (Yachi et al. 2012). Simultaneous labelling by the two toxins with a green-fluorescent-protein tag showed that the plasma membrane displayed heterogeneous SM pools. Furthermore, in permeabilised cells, lysenin exclusively stained late endosomes among intracellular organelles, whereas EqtII also stained recycling endosomes. Intracellular SM detection by EqtII was abolished by a sphingomyelin synthase 1 (SMS1) knockdown, but not by an SMS2 knockdown, providing further evidence for the existence of distinct SM populations. These observations were later refined (Makino et al. 2015), suggesting that both the SM content and membrane distribution are crucial for pathophysiological events, thus opening the door to therapeutic interventions targeting the role of the different SM membrane pools. Almost one decade later, this model was further refined and expanded to the cytosolic leaflet of the plasma membrane, using non-toxic modified versions of EqtII (NT-EqtII) (Mori et al. 2024). This NT-EqtII, resulting from two-point mutations (L26A and P81A), retained the specificity and affinity to SM of WT-EqtII. The non-toxic SM probe allowed us to show the presence of SM in the cytosolic leaflet of the plasma membrane of a variety of cells. It also showed that SM forms small, transient clusters with typical raft lipids such as cholesterol in the cytosolic leaflet, providing a new framework for future studies on signal transduction.

## Biotechnological uses of nanopores

Nanopores are emerging as fundamental for the biotechnological design of countless devices. Many different approaches and materials are being employed to design useful tools of nanometric dimensions. PFPs stand out as molecular champions in building nanopores and, together with recent advances in synthetic biology and protein engineering, have opened up new possibilities in fields such as drug delivery (Tabata et al. 2012), cell-based therapies, including immunotherapy (Cao et al. 2025), or biosensing (Anderluh and Lakey 2008; Fahie et al. 2015). In fact, PFPs are also at the heart of cutting-edge technology that seeks to optimise the use of DNA as a computational storage material. Individual readings of the sequence of single DNA strands are essential for decrypting the stored information (Takiguchi et al. 2025). This technology is being developed at such a pace that low-cost portable devices, such as *Oxford Nanopore Technologies MinION*, are now available. These devices, based on membrane nanopores (Zhang et al. 2022) made of modified PFPs, have already been described in the literature (Ying et al. 2022) for purposes such as single-molecule sequencing of nucleic acids (DNA or RNA) (Stoddart et al. 2009; Wloka et al. 2016), peptides identification and protein sequencing (De Lannoy et al. 2021; Huang et al. 2019; Lucas et al. 2021a, 2021b), or the discrimination of protein glycosylations (Versloot et al. 2022).

In addition to PFP-based systems, DNA-based devices are being developed to construct nanopore molecular tools (Yang et al. 2024). These include constructs that combine protein nanopores with DNA aptamers (Mohammad et al. 2012; Soskine et al. 2012), or a DNA-made light-controlled nanopore which, upon irradiation, opens for transporting molecular cargo across membranes in a non-invasive manner (Offenbartl-Stiegert et al. 2022). Therefore, biological nanopore technology emerges as a powerful strategy for single-molecule analysis of peptides and nucleic acids (Akeson et al. 1999; Bush et al. 2017; Kasianowicz et al. 1996; Wang et al. 2013), including sequencing or detection of chemical modifications (Dorey and Howorka 2024; Versloot et al. 2022). So far, as stated above, membrane-spanning nanopores are widely used in commercial nucleic acid sequencing devices, and they show strong potential for protein sequencing (Motone et al. 2024) and metabolite detection (Deamer et al. 2016; Dorey and Howorka 2024; Wang et al. 2021). These methods rely on monitoring ionic current changes as molecules translocate through the nanopore under an applied electric field, allowing discrimination of biomolecules, including nucleic acids, proteins, and complexes, based on their size, while event frequency provides an estimate of their concentration. A single-molecule nanopore sequencing is reagent-free and requires a cheap electrical supply (Branton et al. 2008). A couple of decades ago, it was estimated that a 10,000-protein nanopore device could potentially determine a human genome sequence within less than a day (Bayley 2006; Branton et al. 2008). A working time frame that is being quickly approached. A major limitation of these devices is, however, non-specific absorption of proteins to the nanopore surface, which may lead to pore clogging. This problem can be overcome by using a wide variety of designs (Stoddart et al. 2014) or by the incorporation of protein or nucleic acid adaptors (Stoddart et al. 2009).

The staphylococcal α-hemolysin (α-HL) protein nanopore is paradigmatic. α-HL has been thoroughly investigated as a cheap and fast nanopore to perform all these biotechnological tasks. There are examples of their use for covalent chemistry at the single-molecule level (Aksimentiev and Schulten 2005; Luchian et al. 2003a, 2003b), nucleic acid analysis and sequencing (Akeson et al. 1999; Japrung et al. 2010; Kasianowicz et al. 1996; Maglia et al. 2008), or proteomic analysis, including resolution of post-translational modifications (Wei et al. 2025). α-HL produces a β-PFP mushroom-shaped pore, with a constriction of 1.4 nm in diameter that divides the interior of the pore into the vestibule and the barrel compartments (Song et al. 1996) (Fig. 1). When an electrophoretic *cis*-to-*trans* translocation of ssDNA through its pore is performed, a blockade of the ionic current occurs. The magnitudes of these blockades and the translocation times are different for polynucleotides of various compositions. This led to the development of fast, inexpensive methods for sequencing ssDNA that identify bases individually at specific sites within the ssDNA as the DNA moves through the pore (Branton et al. 2008). However, this is only possible if the DNA is immobilised within the pore (Purnell and Schmidt 2009; Stoddart et al. 2009). Free ssDNA is translocated too rapidly, and then base discrimination is ineffective. Therefore, several methods have been developed to slow the speed at which DNA transverses these pores (Benner et al. 2007; Cockroft et al. 2008). Intramolecular base-pairing leading to secondary structures can also interfere with the sequencing process (DeGuzman et al. 2006; Lakatos et al. 2005). Fortunately enough, these interactions can be avoided simply by using high denaturant concentrations, as the pore remains functional even at 7 M urea (Japrung et al. 2010).

Nanopore sensing, which was initially widely applied for single-molecule detection of nucleic acids, is now being also extended to protein sensing (Wei et al. 2025; Yamaji et al. 2025). Using again the α-HL nanopore, several authors have shown that a β-hairpin peptide can be captured via electrophoretic approaches, exhibiting long dwell times within the nanopore and leading to multiple current blockade levels. However, in these experiments, the readout is more complex than for DNA. The peptide tested showed non-sequential transitions among four distinct blockade levels, indicating that peptide dynamics in nanopores could not be modelled along a single reaction coordinate. More recently, it has been shown that peptides with acetylation, phosphorylation, citrullination replacement of arginine, or β-hydroxybutyrylation modification, including isomeric peptides, can be separated from each other and discriminated in a series of different mixtures (Wei et al. 2025). There is even an example of how a modular engineered α-HL nanopore can function as GroES that, in complex with GroEL, forms a two-stroke protein-folding nanomachine acting as a transmembrane co-chaperonin (Ho et al. 2015).

Independently of its innate cytotoxicity against many eukaryotic cells, the ability of α-HL to generate pores in the plasma membrane has also sparked interest because of other potential therapeutic and biotechnological applications (Chandramouli et al. 2016; Cimpanu et al. 2025; Guan et al. 2025; Krishnasastry et al. 1994; Panchal et al. 2002). For example, its use as an agent against some tumoural cells has been not only considered but studied to some detail (Bayley 1994). These studies relied on genetic engineering to modify pore specificity and introduced the possibility of turning on and off the pore by means of the employment of a series of activators and inhibitors of different nature (Krishnasastry et al. 1994). The goal was to facilitate the penetration of proteases that would act on specific cancer cells. α-HL was also considered as a potential facilitator for drug delivery.

In recent years, the potential of PFPs as catalytic nanomachines has also been under investigation. This mechanism involves, for example, modification of protein residues to transform the pore into a nanoreactor (Ho et al. 2015; Robles-Martín et al. 2023). A good example of this approach is the creation of an α-HL nanopore that acts as an ATPase, a possibility which is already being studied (Ho et al. 2015), or the much more recent use of α-HL as a sensitive peptide discrimination nanopore (Wei et al. 2025). In summary, α-HL nanopore is a paradigmatic and thoroughly investigated system as a cheap and fast nanopore version to perform biotechnological tasks such as covalent chemistry at the single-molecule level (Aksimentiev and Schulten 2005; Luchian et al. 2003a, b), nucleic acid analysis and sequencing (Akeson et al. 1999; Japrung et al. 2010; Kasianowicz et al. 1996; Maglia et al. 2008; Stoddart et al. 2009), or proteomic analysis (Wei et al. 2025).

## Biotechnological modification of actinoporin’s nanopores

**Fig. 4:**
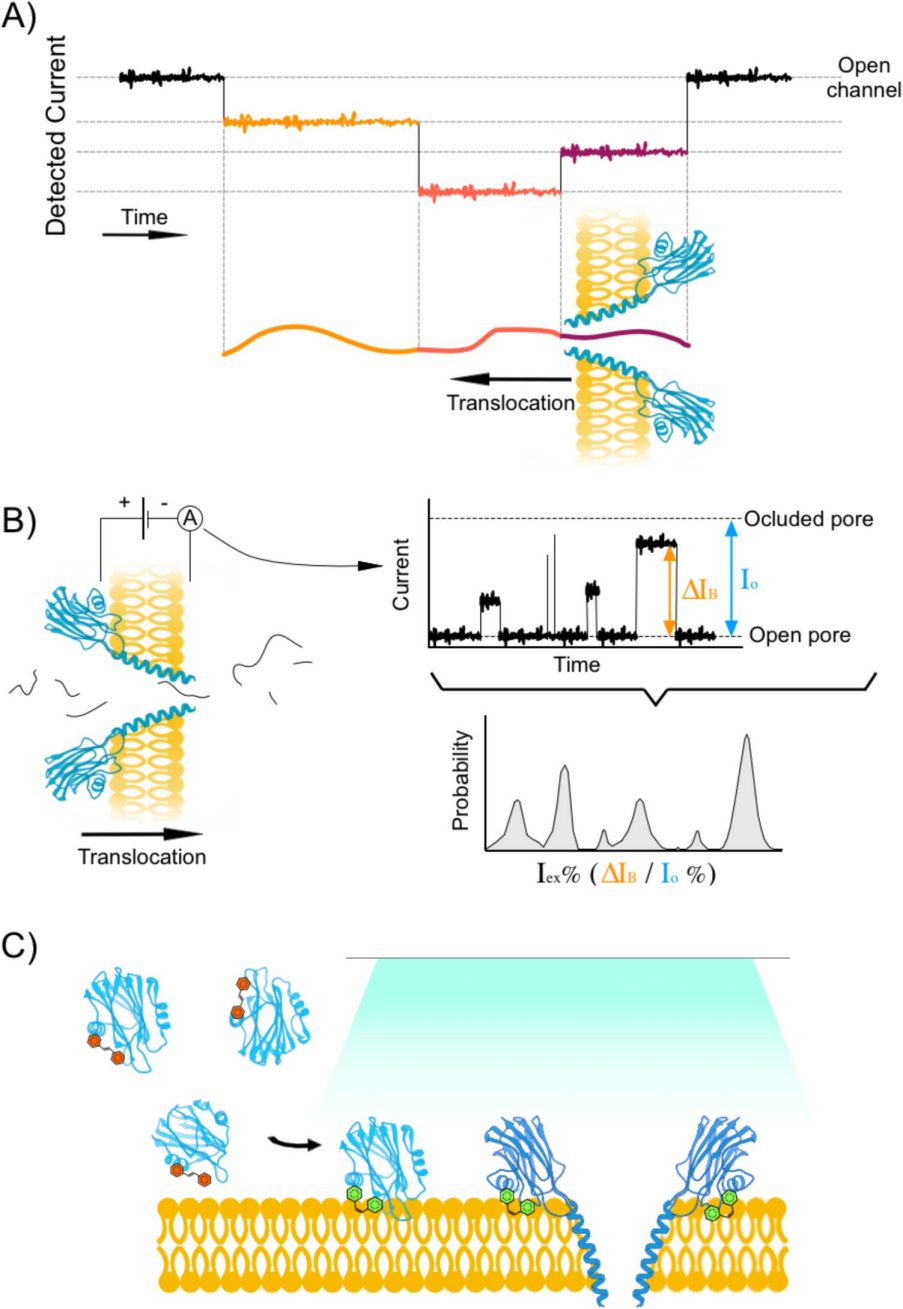
Biotechnological uses of actinoporins. **A** Actinoporin-based DNA sequencing. The current detected across the pore depends upon the sequence of the DNA that is being translocated. Adapted from Wlocka *et al.* 2016. **B** Actinoporin-based proteomic profiling. Digested peptides are translocated across an actinoporin pore. The excluded current (I_ex_), which relates to the volume of the translocated peptide, is calculated as the fraction of blocked current (∆I_B_) over the current for the open pore (I_o_): I_ex_ % = ∆I_B_ / I_o_ %. The resulting histogram can be used for protein identification. Adapted from Lucas *et al.* 2021b. **C** Light-controlled activation of actinoporin activity. Activity can be controlled by the addition of an azobenzene derivative. The *trans* isomer (depicted with dark-orange-filled cycles) blocks the binding pocket of the modified actinoporin. Exposure to the appropriate wavelength causes the isomerization of the azobenzene derivative into its *cis* isomer (depicted with green-filled cycles), unlocking the binding site and activating the actinoporin. Adapted from Volarić *et al.* 2024

The first attempt to modify an actinoporin molecule in order to create a biotechnologically useful pore of nanometrical size was described by Maglia’s group about one decade ago (Wloka et al. 2016). They engineered FraC for ssDNA sequencing (Fig. 4A), constructing a modified pore capable of distinguishing among homopolymeric C, T, and A polynucleotide stretches. They also verified the unexpected result that dsDNA could translocate through the nanopore at high applied potentials, in spite of the narrow ~ 1.2-nm *trans* constriction of the FraC pore lumen. They explained this observation through the deformation of the α-helical transmembrane region of the pore. Some years later, the same group developed engineered versions of FraC to produce biological nanopores capable of analysing and discriminating a wide range of peptides of different lengths, allowing direct readouts of the mass of single peptides in solution (Huang et al. 2017, 2019; Lucas et al. 2024; Mutter et al. 2021).

Interestingly, nanopores of different widths can be obtained by altering the protein oligomeric composition after mutating key residues for the interaction between the nanopore and the lipid interface (Huang et al. 2019). Using this procedure, three distinct nanopore populations were obtained, composed of 8, 7, or 6 protomers. These displayed different inner width diameters at the narrowest opening end of the pore, located at the *trans* end of the ensemble. When characterised in lipid bilayers, all three nanopore types showed well-defined single conductance distributions, a steady open pore current, and comparable power spectra. These nanopores were then able to accommodate peptides ranging from 22 to 4 amino acids in length. Smaller peptides could be detected using further fine tuning of the transmembrane region of the nanopore (Huang et al. 2019), and by introducing amino acids with bulky side chains in the recognition volume of the nanopore. It was also shown how the ionic signal detected was pH-dependent, only varying with the mass of the peptide irrespective of its sequence when the pH was 3.8. At higher pH values, however, the current signal of negatively charged peptides was higher than expected from their mass alone (Huang et al. 2019). Introducing aromatic amino acids at precise positions within the lumen of α-helical FraC nanopores increases the capture frequency of peptides, largely improving the discrimination among peptides of similar size (Lucas et al. 2021a, b). Current efforts aim to improve the recognition and capture rate of peptides by nanopores. This would facilitate the development of a real-time, single-molecule analyser for peptide recognition and identification (Fig. 4B). Actinoporin oligomers of different stoichiometries have been detected not only for FraC, but also for StnI and II, both in the absence (De los Ríos et al. 1999; García-Linares et al. 2022; Huang et al. 2019) and in the presence (Arranz et al. 2025) of lipid bilayers. Not surprisingly, these results show that actinoporins can be engineered into ready-to-use nanopore detectors, eventually integrated into protein sequencing and proteomic detection systems.

A significant step forward in this direction was the publication in 2019 of a photoswitchable version of FraC (Mutter et al. 2019). The switch consisted in an azobenzene covalently bound in the proximity of one of the SM binding pockets of this actinoporin (Arranz et al. 2025; Tanaka et al. 2015b). Mutation of strategically located amino acids into cysteine (absent in actinoporins; see above) facilitated the construction of the azobenzene derivative. The rationale was to obtain a FraC mutant that would not bind the membrane if the azobenzene was in the *trans* configuration, as the charged group in the *para* position was expected to limit the affinity with the hydrophobic bilayer. The azobenzene could then be reversibly switched to the *cis* state upon illumination, allowing binding to the membrane and the subsequent pore formation. As designed, one of the various constructs assayed was completely inactive but induced full lysis of cultured cancer cells upon irradiation with near UV-light. This selective irradiation also allowed isolation of individual nanopores in artificial lipid membranes (Mutter et al. 2019).

Regarding potential therapeutic uses, irradiation with UV light is not ideal, mainly because of its limited penetration depth and well-known potential health issues. The same research group of authors reported, 5 years later, a modified version of their system that was controlled using visible light (Volaric et al. 2024). This new construct relied on a visible-light-operated tetra-orthofluoro-azobenzene photoswitch. This new mechanism is based on a nucleophilic aromatic substitution reaction for installing a solubilising sulfonate group onto the electron-poor photoswitch structure. This version of the azobenzene was again attached to two cysteine mutants of FraC, and their respective activities were evaluated on red blood cells. In both mutants, the green-light-irradiated sample, containing now the *cis*-azobenzene isomer, was more active compared to the blue-light-irradiated sample, which did not properly bind to the bilayer (Fig. 4C). However, finding a different trigger to regulate these nanopores remains challenging since the penetration power of visible light is still poor for therapeutic uses.

Altogether, protein nanopores have become versatile tools for identifying biomolecules thanks to their ability to characterise individual molecules without the need for labelling. However, to the best of our knowledge, so far they have not been widely used in the design of new artificially designed enzymes. In this regard, a very significant breakthrough was made through the combined use of structure-based computational modelling tools and different experimental techniques, as explained in the next section.

## Actinoporin nanopores as a potential solution for microplastic contamination

Microplastics are pervasive in the food we eat, the water we drink, and the air we breathe. For this reason, a severe global health problem stems from the ubiquitous presence of micro- and, most importantly, nanoplastic particles. These pollutants are almost impossible to remove, precisely because of their minuscule size (Gigault et al. 2018). They are indeed capable of integrating into and intoxicating cells and tissues of all types of living beings, including humans, mostly through the food chain. In agreement with these assertions, recent studies have confirmed the presence of micro- to nanoplastics equivalent to trillions of particles in the air of some cities (Allen et al. 2019), in mineral water (Schymanski et al. 2021), in Alpine surface snow, and even in ice samples from protected areas such as Antarctica (Materic et al. 2022a, b; Yang et al. 2021). The ubiquity of plastic and plastic debris is thus causing an unprecedented ecological crisis that is affecting all living forms, from macro- to microorganisms (Rosenboom et al. 2022; York 2020). This cumulative environmental burden of plastic waste is increasingly becoming an irreversible global catastrophe (Yan 2022). Paradoxically, plastic is also a valuable raw material. Therefore, recycling is a promising, low-CO_2_ releasing alternative to incineration, either as a basis for polymer synthesis or as a carbon source for fermentation (Sadler and Wallace 2021).

Hence, it seems mandatory to incorporate extra efforts to expand the toolbox for recycling plastic mixtures. Researchers have been exploring both chemical and biological processes to develop alternative recycling routes capable of converting plastic waste into commercially valuable chemicals (Sullivan et al. 2022). For example, over the past 3 years polystyrene has been converted back to styrene monomers in the presence of table salt and an oxidised copper scrubber (Kumar et al. 2022). Using alumina-supported platinum nanoparticles, polyethylene was turned into long-chain alkyl aromatics, which have broad uses as surfactants, lubricants, refrigeration fluids, and more (Zhang et al. 2020). Finally, enzymes to convert PET back into its monomers, forming a closed-loop recycling process, have also been developed (Lu et al. 2022). In this context, we considered that PFPs could be potential candidates for creating new biocatalytic tools to eliminate this kind of pollutants. PFPs would provide additional advantages over existing systems, such as improved efficiency and the possibility of performing catalytic activity at, comparatively, low temperatures (Robles-Martín et al. 2023). In that work, it was demonstrated that biocatalytic versions of these nanopores are not only possible but can be designed to break down different types of primary polyethylene terephthalate of submicrometric and nanometric sizes (nPET), using an engineered version of FraC. In fact, two different nanoreactors were constructed. They showed many advantages over other current methods, such as low reaction temperatures (between 30 and 40 °C), activity in moderate pH ranges (around 7.0), high stability, biodegradability, and absence of toxicity to living organisms (Robles-Martín et al. 2023; Wei and Bornscheuer 2023). The results, compiled in the corresponding article (Robles-Martín et al. 2023), constitute a proof of concept demonstrating that the approach is feasible, though they only represent the starting point for future improvements.

**Fig. 5:**
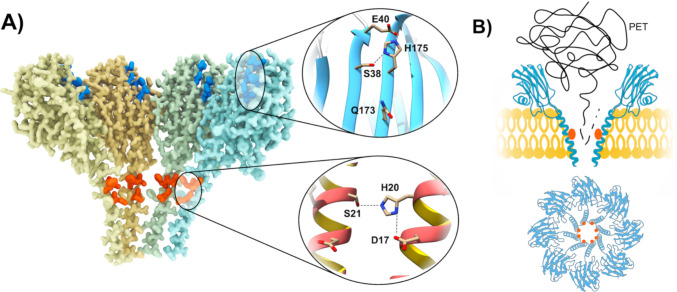
Catalytic centres of the FraC-based nanoreactor. **A** Half-pore view of a FraC pore (PDB ID: 9GKL) in which the incorporated catalytic centres are indicated in red and blue (Arranz et al. 2025; Robles-Martín et al. 2023). Close-up views of the catalytic centres in the β-sandwich (dark blue) and in the α-helices (red) are shown, indicating the residues involved. The centre located in the helices is shared between subunits, with the S21 residue being part of the adjacent monomer within the pore. **B** Schematic representation of the PET-digestion by the nanoreactors. Red dots indicate the catalytic sites. The picture at the bottom, showing a top-down view of the structure, highlights the spatial concentration of the catalytic centres, which favours the progress of the reaction. Graphs and images were built using UCSF Chimera (Pettersen et al. 2004)

In that work, the entire inner surface of the wild-type FraC pore structure was explored using the Protein Energy Landscape Exploration (PELE) software (Alonso et al. 2020; Roda et al. 2022). The selected probes were three esters commonly hydrolysed by most esterases and lipases (Alonso et al. 2020). The results of this analysis suggested the introduction of two separate and different catalytic triads within the FraC actinoporin pore. One was located at the α-helices penetrating the membrane core and another one at the funnel-shaped corona of the pore (Fig. 5). Hence, two different versions of a multicatalytic (eight catalytic sites per pore) PFP-based enzyme were produced. These constructs were able to depolymerise PET nanoparticles in the 30–40-°C temperature range, competing in efficacy with most known PETase enzymes (Ahituv et al. 2025; Bell et al. 2022; Cui et al. 2021; Lu et al. 2022; Son et al. 2019; Tournier et al. 2020; Turak et al. 2025; Vidal et al. 2024; Yoshida et al. 2016; Zhang et al. 2024). Another advantage of these newly designed catalytic nanopore proteins was that the two variants generated different products. One variant broke down PET particles more thoroughly, thus being useful for degradation of nPET in wastewater treatment plants. Interestingly, the products of the activity of the other enzyme were the initial components needed for recycling (Robles-Martín et al. 2023).

## Future developments

Knowledge on petroleum-based plastics enzymatic degradation is still sparse (Buchholz et al. 2022). For example, the most abundant plastic, PET, is the main component in many synthetic fibres and water bottles. Nevertheless, it only comprises about 5.0% of the total identified plastic particles, accounting for 14.4% of total plastic waste (Cabernard et al. 2022; Geyer et al. 2017; Rosenboom et al. 2022; York 2020). Then, PET is just an example of plastic waste. Another example is polyethylene, the most produced plastic today, whose different density variants have been used in an ample variety of products. There is also polystyrene, usually foamy and one of the most widely used plastics to make containers, lids, bottles, trays, or tumblers. Not to forget polyamide, the basis of nylon and extensively used to manufacture textile fabrics (Yang et al. 2021). Finally, polyurethane is another extensively distributed product, mostly as part of many foamy protective coats. Unfortunately, the numerous advantages offered by these non-easily degradable plastics, such as versatile physical properties and low manufacturing costs, have yet to be meaningfully challenged by any other material in the marketplace.

Currently, less than 50 verified plastics-active enzymes have been described (Buchholz et al. 2022). Examples acting on the polymers PET and polyurethane have been reported, together with a detailed biochemical and structural description. Still, very few polyamide-active (nylon-active) enzymes are known (Buchholz et al. 2022; Negoro et al. 2012). Interestingly, PET and polyamide are the main plastic targets of our research.

Biological nanopores hosting rather narrow lumen channels, such as the aforementioned nanoreactor PETase (Robles-Martín et al. 2023) (Fig. 5), are revolutionising single-molecule analytics, yet they remain constrained in resolution and analyte scope. Unfortunately, micro- and even nanoplastic particles are much bulkier than the width of the pores so far studied for single-molecule analytical studies. As early as 2004, it was demonstrated how CDC pores of around 30 to 50 monomers could be gathered together to produce a complete ring complex which facilitates the insertion of β-barrel and leads to the creation of a 25–30-nm-diameter functional pore complex (Czajkowsky et al. 2004). They are part of the group of large β-PFPs (Johnstone et al. 2025; Liu et al. 2025a; Xia et al. 2021). Enlarged β-barrel assemblies might be a promising solution for the more efficient degradation and recycling of plastic submicro- and nanoparticles. For example, using a secretin from the acidophile *Acidithiobacillus caldus*, it has been demonstrated that it is possible to build a 14-mer nanopore scaffold with exceptional membrane-insertion capacity (Liu et al. 2025b). Modified versions of this nanopore have shown exceptional capacities to characterise homopolynucleotides and proteins at the single-molecule level. Similarly, a nanopore formed by the complement component 9 (C9) has yielded an exceptionally large pore of 10 nm width. These nanopores have shown the ability to distinguish among a wide range of protein sizes and conformations based on differences in current modulations within resistive pulses and the corresponding differences in approximations of their shape (Chanakul et al. 2025). Reconstitution of poly(C9) nanopores into lipid mimetics, such as amphipols, enabled highly sensitive and accurate characterisation of a wide range of natively folded proteins on a single-molecule level.

**Fig. 6:**
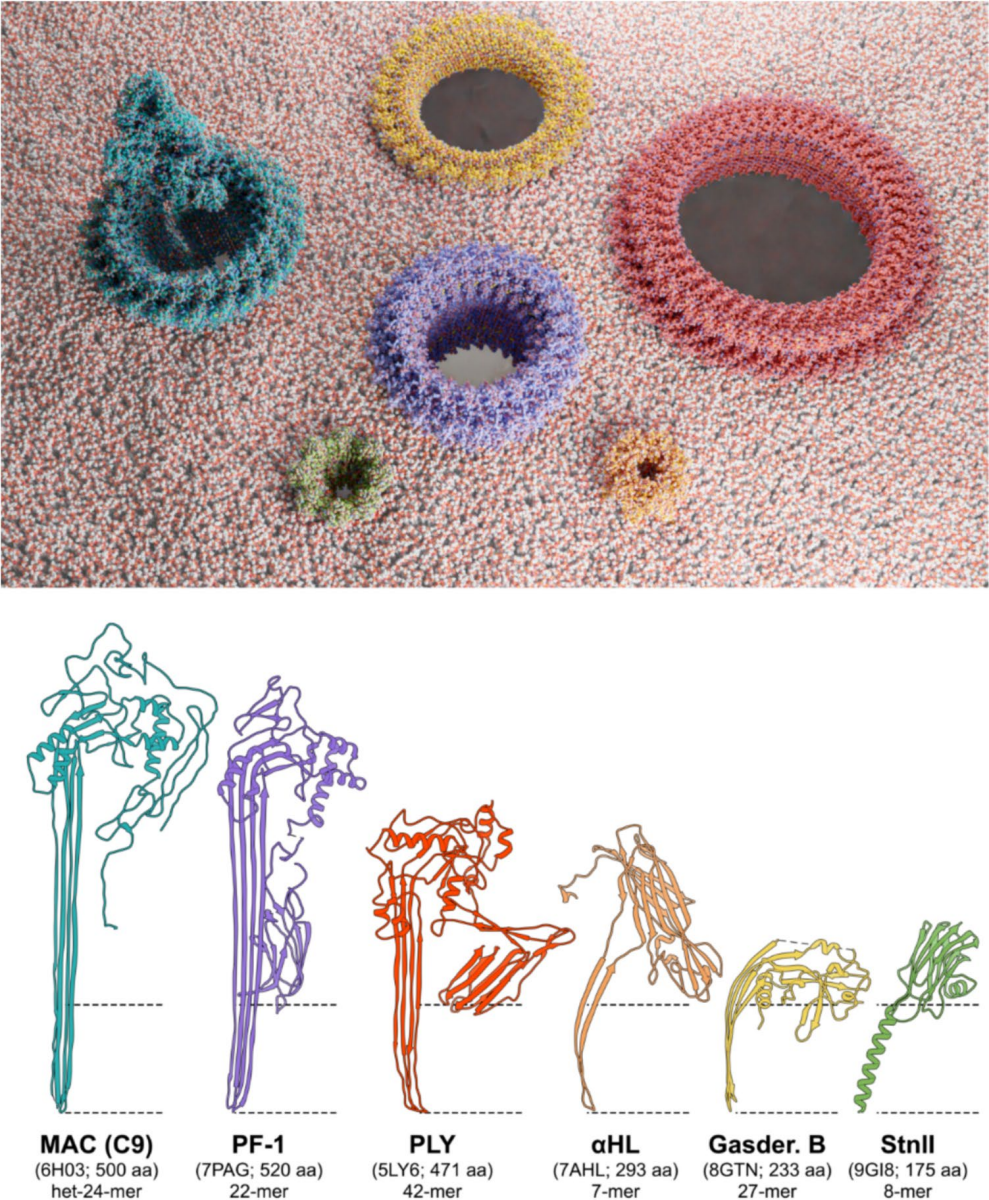
Size comparison of different pores and their protomers. Top: artistic all-atom representation of five β-PFP pores and the StnII pore in a membrane. Shown are the hetero-24-mer of the human membrane attack complex (MAC, in blue; 6HO3) (Menny et al. 2018), the 27-mer of the human gasdermin-B pore (in yellow; 8GTN) (Zhong et al. 2023), the 22-mer of the murine lymphocyte perforin-1 (PF-1, in lilac; 7PAG) (Ivanova et al. 2022), the 42-mer of *S. pneumoniae*’s pneumolysin (PLY, in red; 5LY6) (Van Pee et al. 2017), the 7-mer of *E. coli*’s α-HL (in orange; 7AHL) (Song et al. 1996), and the 8-mer of *S. helianthus’* StnII (in green; 1GWY) (Mancheño et al. 2003). The MAC complex consists of 18 C9 subunits, plus one chain of each of the following proteins: C5, C6, C7, C8α, C8β, and C8γ. Graphs and images were built using UCSF Chimera (Pettersen et al. 2004). Pores of PF-1 and PLY were constructed using UCSF Chimera X and applying C22 and C42 symmetries for an approximate inner pore diameter of 110 Å and 250 Å, respectively. StnII and α-HL pores are shown for scale. Bottom: protomers of the pores shown above, in their pore conformation. The PDB ID and the number of residues per chain are also indicated in brackets. Only a C9 subunit is shown for the MAC complex. The broken lines loosely indicate the estimated position of the membrane. Images were built using UCSF Chimera and its different extended versions (Goddard et al. 2005; Meng et al. 2023; Pettersen et al. 2004). Blender (Team BD 2023) and Molecular Nodes Zenodo (Johnston et al. 2025) have also been used to build these images

Another recent example of a very similar approach is the self-assembled PLY pores, a CDC from *Streptococcus pneumoniae*. PLY forms a stable transmembrane pore with a diameter of about 20 nm which presents an excellent low noise when tested in nanopore-based resistive pulse recordings (Mukhopadhyay et al. 2025). Its exceptionally large pore diameter enables the characterisation of the size and shape of individual proteins as well as aggregated complexes, as demonstrated by following the time course of the formation of oligomers of tau protein in solution (Mukhopadhyay et al. 2025). Accordingly, the possibility of obtaining plastic-degrading nanopores of wider lumen diameters is worth exploring. These nanoreactors would not only provide the possibility of degrading larger plastic particles but also would provide a much higher number of catalytic sites per pore, given the large number of protomers (Fig. 6). Most likely, once this objective has been achieved, a carefully considered mixture of pores of different diameters and different catalytic specificities will work much better in reducing micro- and nanoplastic waste than the potential use of simply a homogeneous type of modified PFP.

One-pot cascade reactions are chemical processes highly appealing to the industrial sector, as they allow the synthesis of complex products starting from relatively simple reaction conditions. Performing a cascade reaction within a single enzyme introduces complexities and uncertainties. Therefore, the study and design of either biocatalysts performing different chemical reactions in the same protein scaffold or by linking multiple domains have become a hot topic in protein engineering through a number of strategies. Another desirable development would be to ease the production and purification, as well as enhance the stability, of engineered plastic-degrading catalytic nanopores. These nanopores will be able to act as both filtering systems and reaction chambers, as new alternatives for cleaning, recycling, and upcycling of micro- to nanosized plastic particles. Large β-PFPs would be an ideal platform to include multiple catalytic sites with different but complementary activities and so construct multitask nanoreactors capable of degrading plastic particles of different sizes and chemical compositions. This concept is gradually gaining recognition/relevance under the name of PluriZymes (Robles-Martín et al. 2024; Roda et al. 2022; Vidal et al. 2024). This group of artificially engineered multitask enzymes, which rely on inserting extra active sites, allows the inclusion of different chemistries in a single protein scaffold, reducing the costs and optimisation of protein expression. Thus, PluriZymes may become part of a next generation of nanopore-based biocatalysts with a wide range of applications, including industrial processes. This repertoire could be further expanded to a next generation of nanopores that might also be fabricated using unnatural amino acids that hold a negative charge at a low pH range (e.g., sulphate or phosphate groups). Alternatively, peptides might be chemically modified (e.g., by esterification) to neutralise the negative charge (Huang et al. 2019). Overall, this methodology would be highly interesting in order to solve the increasingly problematic issue of treating residual waters from the textile industry.

The possibility of producing transmembrane nanopores at the surface of specific cells (mostly bacteria), or even on membrane analogues, also opens the possibility for the design of microbial reactors with integrated catalytic pores supporting multiple conversions, yet to be defined. Within this idea, it has very recently been published a revolutionary *proof-of-concept* study for reprogramming a common strain of *E. coli* to degrade PET nanoparticles without introducing foreign DNA, nor compromising native cellular fitness (Vidal et al. 2025). An article that is most likely showing us the way forward to follow in the field of waste and contaminants disposal and recycling.

## Conclusions

PFPs are proving to be ideal platforms for the development of multiple biotechnological tools. Its wide range of potential applications varies from DNA and peptide sequencing to the introduction of new enzymatic activities, such as the aforementioned PETase based on actinoporin FraC, whose nanopore has been transformed into two different nanoreactors capable of degrading PET. In fact, actinoporins in particular, given their relatively simple size and structure, and PFPs in general, combined with the development of state-of-the-art software, allow scientists to glimpse a new horizon of functionalities and applications that could help address the environmental challenges facing humanity today, such as the overwhelming and growing pollution from plastic particles.

## Data Availability

No datasets were generated or analysed during the current study.

## Abbreviations

α-HL: α-Hemolysin
C9: Complement component C9
CDC: Cholesterol-dependent cytolysins
Chol: Cholesterol
NT-EqtII: Non-toxic modified version of EqtII
PELE: Protein Energy Landscape Exploration
PET: Polyethylene terephthalate
PFP: Pore-forming protein
PFO: Perfringolysin O
PLY: Pneumolysin
SM: Sphingomyelin
PF-1: Perforin-1
SMS: Sphingomyelin synthase

## References

[CR1] Ahituv N, Freund D, Mireles R, Noda-Garcia L (2025) The diversity of PET degrading enzymes: a systematic review of sequence, structure, and function. Protein Sci 34:e70282. 10.1002/pro.70282 40944418 10.1002/pro.70282 PMC12432417

[CR2] Akeson M, Branton D, Kasianowicz JJ, Brandin E, Deamer DW (1999) Microsecond time-scale discrimination among polycytidylic acid, polyadenylic acid, and polyuridylic acid as homopolymers or as segments within single RNA molecules. Biophys J 77:3227–3233. 10.1016/S0006-3495(99)77153-5 10585944 10.1016/S0006-3495(99)77153-5 PMC1300593

[CR3] Aksimentiev A, Schulten K (2005) Imaging α-hemolysin with molecular dynamics: ionic conductance, osmotic permeability, and the electrostatic potential map. Biophys J 88:3745–3761. 10.1529/biophysj.104.058727 15764651 10.1529/biophysj.104.058727 PMC1305609

[CR4] Alegre-Cebollada J, Oñaderra M, Gavilanes JG, Martínez-del-Pozo A (2007) Sea anemone actinoporins: the transition from a folded soluble state to a functionally active membrane-bound oligomeric pore. Curr Protein Pept Sci 8:558–572. 10.2174/138920307783018686 18220843 10.2174/138920307783018686

[CR5] Allen S, Allen D, Phoenix VR et al (2019) Atmospheric transport and deposition of microplastics in a remote mountain catchment. Nat Geosci 12:339–344. 10.1038/s41561-019-0335-5

[CR6] Alm I, García-Linares S, Gavilanes JG, Martínez-del-Pozo A, Slotte JP (2015) Cholesterol stimulates and ceramide inhibits sticholysin II-induced pore formation in complex bilayer membranes. Biochim Biophys Acta Biomembr 1848:925–931. 10.1016/j.bbamem.2014.12.017 10.1016/j.bbamem.2014.12.017 25546840

[CR7] Alonso S, Santiago G, Cea-Rama I et al (2020) Genetically engineered proteins with two active sites for enhanced biocatalysis and synergistic chemo- and biocatalysis. Nat Catal 3:319–328. 10.1038/s41929-019-0394-4

[CR8] Anderluh G, Lakey JH (2008) Disparate proteins use similar architectures to damage membranes. Trends Biochem Sci 33:482–490. 10.1016/j.tibs.2008.07.004 18778941 10.1016/j.tibs.2008.07.004

[CR9] Anderluh G, Maček P (2002) Cytolytic peptide and protein toxins from sea anemones (Anthozoa: Actiniaria). Toxicon 40:111–124. 10.1016/s0041-0101(01)00191-x 11689232 10.1016/s0041-0101(01)00191-x

[CR10] Anderluh G, Pungercar J, Krizaj I, Strukelj B, Gubensek F, Maček P (1997) N-terminal truncation mutagenesis of equinatoxin II, a pore-forming protein from the sea anemone *Actinia equina*. Protein Eng 10:751–755. 10.1093/protein/10.7.751 9342140 10.1093/protein/10.7.751

[CR11] Anderluh G, Barlič A, Krizaj I, Menestrina G, Gubensek F, Maček P (1998) Avidin-FITC topological studies with three cysteine mutants of equinatoxin II, a sea anemone pore-forming protein. Biochem Biophys Res Commun 242:187–190. 10.1006/bbrc.1997.7944 9439633 10.1006/bbrc.1997.7944

[CR12] Anderluh G, Barlič A, Podlesek Z et al (1999) Cysteine-scanning mutagenesis of an eukaryotic pore-forming toxin from sea anemone: topology in lipid membranes. Eur J Biochem 263:128–136. 10.1046/j.1432-1327.1999.00477.x 10429196 10.1046/j.1432-1327.1999.00477.x

[CR13] Anderluh G, Krizaj I, Strukelj B, Gubensek F, Maček P, Pungercar J (1999) Equinatoxins, pore-forming proteins from the sea anemone *Actinia equina*, belong to a multigene family. Toxicon 37:1391–1401. 10.1016/s0041-0101(99)00082-3 10414864 10.1016/s0041-0101(99)00082-3

[CR14] Anderluh G, Gokce I, Lakey JH (2004) A natively unfolded toxin domain uses its receptor as a folding template. J Biol Chem 279:22002–22009. 10.1074/jbc.M313603200 15004032 10.1074/jbc.M313603200

[CR15] Arranz R, Santiago C, Masiulis S et al (2025) Elucidating the structure and assembly mechanism of actinoporin pores in complex membrane environments. Sci Adv 11:eadv0683. 10.1126/sciadv.adv0683 40991702 10.1126/sciadv.adv0683 PMC12459404

[CR16] Athanasiadis A, Anderluh G, Maček P, Turk D (2001) Crystal structure of the soluble form of equinatoxin II, a pore-forming toxin from the sea anemone *Actinia equina*. Structure 9:341–346. 10.1016/s0969-2126(01)00592-5 11525171 10.1016/s0969-2126(01)00592-5

[CR17] Bakrač B, Kladnik A, Maček P, McHaffie G, Werner A, Lakey JH, Anderluh G (2010) A toxin-based probe reveals cytoplasmic exposure of Golgi sphingomyelin. J Biol Chem. 10.1074/jbc.M110.105122 20463009 10.1074/jbc.M110.105122 PMC2903383

[CR18] Barlič A, Gutiérrez-Aguirre I, Caaveiro JM, Cruz A, Ruiz-Argüello MB, Pérez-Gil J, González-Mañas JM (2004) Lipid phase coexistence favors membrane insertion of equinatoxin-II, a pore-forming toxin from *Actinia equina*. J Biol Chem 279:34209–34216. 10.1074/jbc.M313817200 15175339 10.1074/jbc.M313817200

[CR19] Barroso RA, Rodrigues T, Campos A, Almeida D, Guardiola FA, Turkina MV, Antunes A (2025) Proteomic diversity of the sea anemone *Actinia fragacea*: Comparative analysis of nematocyst venom, mucus, and tissue-specific profiles. Mar Drugs 23. 10.3390/md23020079 10.3390/md23020079 PMC11857728 39997203

[CR20] Bayley H (1994) Triggers and switches in a self-assembling pore-forming protein. J Cell Biochem 56:177–182. 10.1002/jcb.240560210 7829577 10.1002/jcb.240560210

[CR21] Bayley H (2006) Sequencing single molecules of DNA. Curr Opin Chem Biol 10:628–637. 10.1016/j.cbpa.2006.10.040 17113816 10.1016/j.cbpa.2006.10.040

[CR22] Bell EL, Smithson R, Kilbride S et al (2022) Directed evolution of an efficient and thermostable PET depolymerase. Nat Catal 5:673–681. 10.1038/s41929-022-00821-3

[CR23] Bellomio A, Morante K, Barlič A, Gutiérrez-Aguirre I, Viguera AR, González-Mañas JM (2009) Purification, cloning and characterization of fragaceatoxin C, a novel actinoporin from the sea anemone *Actinia fragacea*. Toxicon 54:869–880. 10.1016/j.toxicon.2009.06.022 19563820 10.1016/j.toxicon.2009.06.022

[CR24] Benner S, Chen RJA, Wilson NA et al (2007) Sequence-specific detection of individual DNA polymerase complexes in real time using a nanopore. Nat Nanotechnol 2:718–724. 10.1038/nnano.2007.344 18654412 10.1038/nnano.2007.344 PMC2507869

[CR25] Branton D, Deamer DW, Marziali A et al (2008) The potential and challenges of nanopore sequencing. Nat Biotechnol 26:1146–1153. 10.1038/nbt.1495 18846088 10.1038/nbt.1495 PMC2683588

[CR26] Buchholz PCF, Feuerriegel G, Zhang H et al (2022) Plastics degradation by hydrolytic enzymes: the plastics-active enzymes database-PAZy. Proteins 90:1443–1456. 10.1002/prot.26325 35175626 10.1002/prot.26325

[CR27] Bush J, Maulbetsch W, Lepoitevin M et al (2017) The nanopore mass spectrometer. Rev Sci Instrum 88:113307. 10.1063/1.4986043 29195372 10.1063/1.4986043 PMC5707180

[CR28] Caaveiro JM, Echabe I, Gutiérrez-Aguirre I, Nieva JL, Arrondo JL, González-Mañas JM (2001) Differential interaction of equinatoxin II with model membranes in response to lipid composition. Biophys J 80:1343–1353. 10.1016/S0006-3495(01)76107-3 11222295 10.1016/S0006-3495(01)76107-3 PMC1301326

[CR29] Cabernard L, Pfister S, Oberschelp C, Hellweg S (2022) Growing environmental footprint of plastics driven by coal combustion. Nat Sustain 5:139–148. 10.1038/s41893-021-00807-2

[CR30] Cao J, Yu Y, Han K et al (2025) Persistent membrane-anchored oligomeric peptides with nanopore formation for targeted immune modulation. Angew Chem Int Ed Engl e202507700. 10.1002/anie.202507700 10.1002/anie.202507700 40932092

[CR31] Chanakul W, Mukhopadhyay A, Awasthi S, Protopopova AD, Ianiro A, Mayer M (2025) Large and stable nanopores formed by complement component 9 for characterizing single folded proteins. ACS Nano 19:5240–5252. 10.1021/acsnano.4c11666 39871506 10.1021/acsnano.4c11666 PMC11823641

[CR32] Chandramouli B, Di Maio D, Mancini G, Brancato G (2016) Introducing an artificial photo-switch into a biological pore: a model study of an engineered α-hemolysin. Biochim Biophys Acta 1858:689–697. 10.1016/j.bbamem.2015.12.030 26744229 10.1016/j.bbamem.2015.12.030

[CR33] Chatterjee A, Naskar P, Mishra S, Dutta S (2025a) Pore formation by pore-forming proteins in lipid membranes: structural insights through cryo-EM. J Membr Biol. 10.1007/s00232-025-00344-5 10.1007/s00232-025-00344-5 40155553

[CR34] Chatterjee A, Roy A, Satheesh T et al (2025) Structural insights into pre-pore intermediates of α-hemolysin in the lipidic environment. Nat Commun 16:6348. 10.1038/s41467-025-61741-x 40640160 10.1038/s41467-025-61741-x PMC12246456

[CR35] Cimpanu A, Park J, Mereuta L, Park Y, Luchian T (2025) Asymmetric nanopore sensing enables single-molecule identification of nucleobases in minimalist peptide nucleic acids. Nano Lett 25:10990–10997. 10.1021/acs.nanolett.5c02717 40577205 10.1021/acs.nanolett.5c02717

[CR36] Cockroft SL, Chu J, Amorin M, Ghadiri MR (2008) A single-molecule nanopore device detects DNA polymerase activity with single-nucleotide resolution. J Am Chem Soc 130:818–820. 10.1021/ja077082c 18166054 10.1021/ja077082c PMC2453067

[CR37] Cosentino K, Ros U, García-Sáez AJ (2016) Assembling the puzzle: oligomerization of α-pore forming proteins in membranes. Biochim Biophys Acta 1858:457–466. 10.1016/j.bbamem.2015.09.013 26375417 10.1016/j.bbamem.2015.09.013 PMC4869852

[CR38] Cui YL, Chen YC, Liu XY et al (2021) Computational redesign of a PETase for plastic biodegradation under ambient condition by the GRAPE strategy. ACS Catal 11:1340–1350. 10.1021/acscatal.0c05126

[CR39] Cyr N (2018) Piercing the lipid raft: the case of *Vibrio cholerae* cytolysin. Biochem J 475:3917–3919. 10.1042/BCJ20180728 30552169 10.1042/BCJ20180728

[CR40] Czajkowsky DM, Hotze EM, Shao Z, Tweten RK (2004) Vertical collapse of a cytolysin prepore moves its transmembrane β-hairpins to the membrane. EMBO J 23:3206–3215. 10.1038/sj.emboj.7600350 15297878 10.1038/sj.emboj.7600350 PMC514522

[CR41] De Lannoy C, Lucas FLR, Maglia G, de Ridr D (2021) *In silico* assessment of a novel single-molecule protein fingerprinting method employing fragmentation and nanopore detection. iScience 24:103202. 10.1016/j.isci.2021.103202 34703997 10.1016/j.isci.2021.103202 PMC8521182

[CR42] De los Ríos V, Mancheño JM, Lanio ME, Oñaderra M, Gavilanes JG (1998) Mechanism of the leakage induced on lipid model membranes by the hemolytic protein sticholysin II from the sea anemone *Stichodactyla helianthus*. Eur J Biochem 252:284–289. 10.1046/j.1432-1327.1998.2520284.x 9580155 10.1046/j.1432-1327.1998.2520284.x

[CR43] De los Ríos V, Mancheño JM, Martínez-del-Pozo A, Alfonso C, Rivas G, Oñaderra M, Gavilanes JG (1999) Sticholysin II, a cytolysin from the sea anemone *Stichodactyla helianthus*, is a monomer-tetramer associating protein. FEBS Lett 455:27–30. 10.1016/s0014-5793(99)00846-7 10428465 10.1016/s0014-5793(99)00846-7

[CR44] Deamer D, Akeson M, Branton D (2016) Three decades of nanopore sequencing. Nat Biotechnol 34:518–524. 10.1038/nbt.3423 27153285 10.1038/nbt.3423 PMC6733523

[CR45] DeGuzman VS, Lee CC, Deamer DW, Vercoutere WA (2006) Sequence-dependent gating of an ion channel by DNA hairpin molecules. Nucleic Acids Res 34:6425–6437. 10.1093/nar/gkl754 17130164 10.1093/nar/gkl754 PMC1702491

[CR46] Ding J, Wang K, Liu W et al (2016) Pore-forming activity and structural autoinhibition of the gasdermin family. Nature 535:111–116. 10.1038/nature18590 27281216 10.1038/nature18590

[CR47] Dorey A, Howorka S (2024) Nanopore DNA sequencing technologies and their applications towards single-molecule proteomics. Nat Chem 16:314–334. 10.1038/s41557-023-01322-x 38448507 10.1038/s41557-023-01322-x

[CR48] Evans JC, Johnstone BA, Lawrence SL, Morton CJ, Christie MP, Parker MW, Tweten RK (2020) A key motif in the cholesterol-dependent cytolysins reveals a large family of related proteins. Mbio 11:e02351-20. 10.1128/mBio.02351-20 32994330 10.1128/mBio.02351-20 PMC7527733

[CR49] Fahie M, Chisholm C, Chen M (2015) Resolved single-molecule detection of individual species within a mixture of anti-biotin antibodies using an engineered monomeric nanopore. ACS Nano 9:1089–1098. 10.1021/nn506606e 25575121 10.1021/nn506606e PMC4958048

[CR50] García-Linares S, Castrillo I, Bruix M, Menéndez M, Alegre-Cebollada J, Martínez-del-Pozo A, Gavilanes JG (2013) Three-dimensional structure of the actinoporin sticholysin I. Influence of long-distance effects on protein function. Arch Biochem Biophys 532:39–45. 10.1016/j.abb.2013.01.005 23376038 10.1016/j.abb.2013.01.005

[CR51] García-Linares S, Rivera-de-Torre E, Morante K et al (2016) Differential effect of membrane composition on the pore-forming ability of four different sea anemone actinoporins. Biochemistry 55:6630–6641. 10.1021/acs.biochem.6b01007 27933793 10.1021/acs.biochem.6b01007

[CR52] García-Linares S, Palacios-Ortega J, Yasuda T, Astrand M, Gavilanes JG, Martínez-del-Pozo A, Slotte JP (2016) Toxin-induced pore formation is hindered by intermolecular hydrogen bonding in sphingomyelin bilayers. Biochim Biophys Acta 1858:1189–1195. 10.1016/j.bbamem.2016.03.013 26975250 10.1016/j.bbamem.2016.03.013

[CR53] García-Linares S, Rivera-de-Torre E, Palacios-Ortega J, Gavilanes JG, Martínez-del-Pozo A (2017) The metamorphic transformation of a water-soluble monomeric protein into an oligomeric transmembrane pore. In: Iglič A, Rappolt M, García-Sáez AJ (eds) Advances in biomembranes and lipid self-assembly, pp 51–97. 10.1016/bs.abl.2017.06.004

[CR54] García-Linares S, Amigot-Sanchez R, Garcia-Montoya C et al (2022) Sticholysin I-II oligomerization in the absence of membranes. FEBS Lett 596:1029–1036. 10.1002/1873-3468.14326 35253212 10.1002/1873-3468.14326

[CR55] García-Ortega L, Alegre-Cebollada J, García-Linares S, Bruix M, Martínez-del-Pozo A, Gavilanes JG (2011) The behavior of sea anemone actinoporins at the water-membrane interface. Biochim Biophys Acta 1808:2275–2288. 10.1016/j.bbamem.2011.05.012 21621507 10.1016/j.bbamem.2011.05.012

[CR56] Geyer R, Jambeck JR, Law KL (2017) Production, use, and fate of all plastics ever made. Sci Adv 3:e1700782. 10.1126/sciadv.1700782 28776036 10.1126/sciadv.1700782 PMC5517107

[CR57] Gigault J, Halle AT, Baudrimont M et al (2018) Current opinion: what is a nanoplastic? Environ Pollut 235:1030–1034. 10.1016/j.envpol.2018.01.024 29370948 10.1016/j.envpol.2018.01.024

[CR58] Goddard TD, Huang CC, Ferrin TE (2005) Software extensions to UCSF chimera for interactive visualization of large molecular assemblies. Structure 13:473–482. 10.1016/j.str.2005.01.006 15766548 10.1016/j.str.2005.01.006

[CR59] González MR, Bischofberger M, Pernot L, van der Goot FG, Freche B (2008) Bacterial pore-forming toxins: the (w)hole story? Cell Mol Life Sci 65:493–507. 10.1007/s00018-007-7434-y 17989920 10.1007/s00018-007-7434-y PMC11131829

[CR60] Guan Z, Sun Y, He Y, Cao J, Tian J, Ji Z (2025) Machine-learning-assisted nanopore sensing solution for the determination of matrix metalloproteinase. Biosens Bioelectron 288:117752. 10.1016/j.bios.2025.117752 40645115 10.1016/j.bios.2025.117752

[CR61] Gupta LK, Molla J, Prabhu AA (2024) Story of pore-forming proteins from deadly disease-causing agents to modern applications with evolutionary significance. Mol Biotechnol 66:1327–1356. 10.1007/s12033-023-00776-1 37294530 10.1007/s12033-023-00776-1

[CR62] Hinds MG, Zhang W, Anderluh G, Hansen PE, Norton RS (2002) Solution structure of the eukaryotic pore-forming cytolysin equinatoxin II: implications for pore formation. J Mol Biol 315:1219–1229. 10.1006/jmbi.2001.5321 11827489 10.1006/jmbi.2001.5321

[CR63] Ho CW, Van Meervelt V, Tsai KC, De Temmerman PJ, Mast J, Maglia G (2015) Engineering a nanopore with co-chaperonin function. Sci Adv 1:e1500905. 10.1126/sciadv.1500905 26824063 10.1126/sciadv.1500905 PMC4730846

[CR64] Hodel AW, Rudd-Schmidt JA, Noori T et al (2025) Acidic pH can attenuate immune killing through inactivation of perforin. EMBO Rep 26:929–947. 10.1038/s44319-024-00365-6 39789387 10.1038/s44319-024-00365-6 PMC11850619

[CR65] Huang G, Willems K, Soskine M, Wloka C, Maglia G (2017) Electro-osmotic capture and ionic discrimination of peptide and protein biomarkers with FraC nanopores. Nat Commun 8:935. 10.1038/s41467-017-01006-4 29038539 10.1038/s41467-017-01006-4 PMC5715100

[CR66] Huang G, Voet A, Maglia G (2019) FraC nanopores with adjustable diameter identify the mass of opposite-charge peptides with 44 dalton resolution. Nat Commun 10:835. 10.1038/s41467-019-08761-6 30783102 10.1038/s41467-019-08761-6 PMC6381162

[CR67] Iacovache I, Bischofberger M, van der Goot FG (2010) Structure and assembly of pore-forming proteins. Curr Opin Struct Biol 20:241–246. 10.1016/j.sbi.2010.01.013 20172710 10.1016/j.sbi.2010.01.013

[CR68] Ivanova ME, Lukoyanova N, Malhotra S, Topf M, Trapani JA, Voskoboinik I, Saibil HR (2022) The pore conformation of lymphocyte perforin. Sci Adv 8:eabk3147. 10.1126/sciadv.abk3147 35148176 10.1126/sciadv.abk3147 PMC8836823

[CR69] Japrung D, Henricus M, Li Q, Maglia G, Bayley H (2010) Urea facilitates the translocation of single-stranded DNA and RNA through the α-hemolysin nanopore. Biophys J 98:1856–1863. 10.1016/j.bpj.2009.12.4333 20441749 10.1016/j.bpj.2009.12.4333 PMC2862201

[CR70] Johnston B, Elferich J, Davidson RB et al (2025) BradyAJohnston/MolecularNodes: v4.5.4. https://zenodo.org/records/17368878. Accessed 20 Oct 2025

[CR71] Johnstone BA, Joseph R, Christie MP et al (2022) Cholesterol-dependent cytolysins: the outstanding questions. IUBMB Life 74:1169–1179. 10.1002/iub.2661 35836358 10.1002/iub.2661 PMC9712165

[CR72] Johnstone BA, Christie MP, Joseph R et al (2025) Structural basis for the pore-forming activity of a complement-like toxin. Sci Adv 11:eadt2127. 10.1126/sciadv.adt2127 40153490 10.1126/sciadv.adt2127 PMC11952106

[CR73] Kasianowicz JJ, Brandin E, Branton D, Deamer DW (1996) Characterization of individual polynucleotide molecules using a membrane channel. Proc Natl Acad Sci U S A 93:13770–13773. 10.1073/pnas.93.24.13770 8943010 10.1073/pnas.93.24.13770 PMC19421

[CR74] Korn V, Pluhackova K (2025) Vastly different energy landscapes of the membrane insertions of monomeric gasdermin D and A3. Commun Chem 8. 10.1038/s42004-024-01400-2 10.1038/s42004-024-01400-2 PMC11802827 39915622

[CR75] Krishnasastry M, Walker B, Braha O, Bayley H (1994) Surface labeling of key residues during assembly of the transmembrane pore formed by staphylococcal α-hemolysin. FEBS Lett 356:66–71. 10.1016/0014-5793(94)01240-7 7988723 10.1016/0014-5793(94)01240-7

[CR76] Kulma M, Anderluh G (2021) Beyond pore formation: reorganization of the plasma membrane induced by pore-forming proteins. Cell Mol Life Sci 78:6229–6249. 10.1007/s00018-021-03914-7 34387717 10.1007/s00018-021-03914-7 PMC11073440

[CR77] Kumar V, Khan A, Rabnawaz M (2022) Efficient depolymerization of polystyrene with table salt and oxidized copper. ACS Sustain Chem Eng 10:6493–6502. 10.1021/acssuschemeng.1c08400

[CR78] Lakatos G, Chou T, Bergersen B, Patey GN (2005) First passage times of driven DNA hairpin unzipping. Phys Biol 2:166–174. 10.1088/1478-3975/2/3/004 16224122 10.1088/1478-3975/2/3/004

[CR79] Lata K, Anderluh G, Chattopadhyay K (2024) Entangling roles of cholesterol-dependent interaction and cholesterol-mediated lipid phase heterogeneity in regulating listeriolysin O pore-formation. Biochem J 481:1349–1377. 10.1042/BCJ20240184 39268843 10.1042/BCJ20240184

[CR80] Lella M, Mahalakshmi R (2017) Metamorphic proteins: emergence of dual protein folds from one primary sequence. Biochemistry 56:2971–2984. 10.1021/acs.biochem.7b00375 28570055 10.1021/acs.biochem.7b00375

[CR81] Leychenko EV, Isaeva M, Tkacheva E et al (2018) Multigene family of pore-forming toxins from sea anemone *Heteractis crispa*. Mar Drugs 16:183. 10.3390/md16060183 29794988 10.3390/md16060183 PMC6025637

[CR82] Liu MJ, Qin XT, Luo ML et al (2025a) Hexameric-based hierarchy in the sizes of a cytolysin pore-forming complex. Biomolecules 15. 10.3390/biom15030424 10.3390/biom15030424 PMC11940705 40149960

[CR83] Liu RH, Zhang K, Feng QS et al (2025) Unveiling acidophilic secretin nanopores for ion exclusion and single-molecule sensing. Adv Funct Mater. 10.1002/adfm.20251725941181574

[CR84] Lu HY, Diaz DJ, Czarnecki NJ et al (2022) Machine learning-aided engineering of hydrolases for PET depolymerization. Nature 604:662. 10.1038/s41586-022-04599-z 35478237 10.1038/s41586-022-04599-z

[CR85] Lucas FLR, Sarthak K, Lenting EM et al (2021) The manipulation of the internal hydrophobicity of FraC nanopores augments peptide capture and recognition. ACS Nano 15:9600–9613. 10.1021/acsnano.0c09958 34060809 10.1021/acsnano.0c09958 PMC8223486

[CR86] Lucas FLR, Versloot RCA, Yakovlieva L, Walvoort MTC, Maglia G (2021b) Protein identification by nanopore peptide profiling. Nat Commun 12. 10.1038/s41467-021-26046-9 10.1038/s41467-021-26046-9 PMC8490355 34608150

[CR87] Lucas FLR, Corstiaan R, Versloot A, Maglia G (2024) Nanopore proteomics. In: Groningen R. Canada: Patent CA 3219470 A1; published Nov 24, 2022. https://patents.google.com/patent/CA3219470A1/en

[CR88] Luchian T, Shin SH, Bayley H (2003) Kinetics of a three-step reaction observed at the single-molecule level. Angew Chem Int Ed Engl 42:1926–1929. 10.1002/anie.200250666 12730970 10.1002/anie.200250666

[CR89] Luchian T, Shin SH, Bayley H (2003) Single-molecule covalent chemistry with spatially separated reactants. Angew Chem Int Ed Engl 42:3766–3771. 10.1002/anie.200351313 12923839 10.1002/anie.200351313

[CR90] Maček P (1992) Polypeptide cytolytic toxins from sea anemones (Actiniaria). FEMS Microbiol Immunol 5:121–129. 10.1111/j.1574-6968.1992.tb05894.x 1384592 10.1111/j.1574-6968.1992.tb05894.x

[CR91] Maček P, Belmonte G, Pederzolli C, Menestrina G (1994) Mechanism of action of equinatoxin II, a cytolysin from the sea anemone *Actinia equina* belonging to the family of actinoporins. Toxicology 87:205–227. 10.1016/0300-483x(94)90252-6 7512761 10.1016/0300-483x(94)90252-6

[CR92] Maglia G, Restrepo MR, Mikhailova E, Bayley H (2008) Enhanced translocation of single DNA molecules through alpha-hemolysin nanopores by manipulation of internal charge. Proc Natl Acad Sci U S A 105:19720–19725. 10.1073/pnas.0808296105 19060213 10.1073/pnas.0808296105 PMC2604925

[CR93] Makino A, Abe M, Murate M et al (2015) Visualization of the heterogeneous membrane distribution of sphingomyelin associated with cytokinesis, cell polarity, and sphingolipidosis. FASEB J 29:477–493. 10.1096/fj.13-247585 25389132 10.1096/fj.13-247585

[CR94] Mancheño JM, Martín-Benito J, Martínez-Ripoll M, Gavilanes JG, Hermoso JA (2003) Crystal and electron microscopy structures of sticholysin II actinoporin reveal insights into the mechanism of membrane pore formation. Structure 11:1319–1328. 10.1016/j.str.2003.09.019 14604522 10.1016/j.str.2003.09.019

[CR95] Marchioretto M, Podobnik M, Dalla Serra M, Anderluh G (2013) What planar lipid membranes tell us about the pore-forming activity of cholesterol-dependent cytolysins. Biophys Chem 182:64–70. 10.1016/j.bpc.2013.06.015 23876488 10.1016/j.bpc.2013.06.015

[CR96] Margheritis E, Kappelhoff S, Cosentino K (2023) Pore-forming proteins: from pore assembly to structure by quantitative single-molecule imaging. Int J Mol Sci 24. 10.3390/ijms24054528 10.3390/ijms24054528 PMC10003378 36901959

[CR97] Martín-Benito J, Gavilanes F, de Los Ríos V, Mancheño JM, Fernández JJ, Gavilanes JG (2000) Two-dimensional crystallization on lipid monolayers and three-dimensional structure of sticholysin II, a cytolysin from the sea anemone *Stichodactyla helianthus*. Biophys J 78:3186–3194. 10.1016/S0006-3495(00)76855-X 10827995 10.1016/S0006-3495(00)76855-X PMC1300900

[CR98] Martínez D, Campos AM, Pazos F et al (2001) Properties of St I and St II, two isotoxins isolated from *Stichodactyla helianthus*: a comparison. Toxicon 39:1547–1560. 10.1016/s0041-0101(01)00127-1 11478962 10.1016/s0041-0101(01)00127-1

[CR99] Martínez D, Otero A, Álvarez C et al (2007) Effect of sphingomyelin and cholesterol on the interaction of St II with lipidic interfaces. Toxicon 49:68–81. 10.1016/j.toxicon.2006.09.019 17113118 10.1016/j.toxicon.2006.09.019

[CR100] Materic D, Holzinger R, Niemann H (2022) Nanoplastics and ultrafine microplastic in the Dutch Wadden Sea - the hidden plastics debris? Sci Total Environ 846:157371. 10.1016/j.scitotenv.2022.157371 35863583 10.1016/j.scitotenv.2022.157371

[CR101] Materic D, Kjaer HA, Vallelonga P, Tison JL, Rockmann T, Holzinger R (2022) Nanoplastics measurements in Northern and Southern polar ice. Environ Res 208:112741. 10.1016/j.envres.2022.112741 35063429 10.1016/j.envres.2022.112741

[CR102] Mechaly AE, Bellomio A, Morante K, González-Mañas JM, Guerin DM (2009) Crystallization and preliminary crystallographic analysis of fragaceatoxin C, a pore-forming toxin from the sea anemone *Actinia fragacea*. Acta Crystallogr Sect F Struct Biol Cryst Commun 65:357–360. 10.1107/S1744309109007064 19342779 10.1107/S1744309109007064 PMC2664759

[CR103] Mechaly AE, Bellomio A, Gil-Carton D, Morante K, Valle M, González-Mañas JM, Guerin DM (2011) Structural insights into the oligomerization and architecture of eukaryotic membrane pore-forming toxins. Structure 19:181–191. 10.1016/j.str.2010.11.013 21300287 10.1016/j.str.2010.11.013

[CR104] Meng EC, Goddard TD, Pettersen EF, Couch GS, Pearson ZJ, Morris JH, Ferrin TE (2023) UCSF ChimeraX: tools for structure building and analysis. Protein Sci 32:e4792. 10.1002/pro.4792 37774136 10.1002/pro.4792 PMC10588335

[CR105] Menny A, Serna M, Boyd CM et al (2018) CryoEM reveals how the complement membrane attack complex ruptures lipid bilayers. Nat Commun 9. 10.1038/s41467-018-07653-5 10.1038/s41467-018-07653-5 PMC6294249 30552328

[CR106] Mohammad MM, Iyer R, Howard KR, McPike MP, Borer PN, Movileanu L (2012) Engineering a rigid protein tunnel for biomolecular detection. J Am Chem Soc 134:9521–9531. 10.1021/ja3043646 22577864 10.1021/ja3043646 PMC3415594

[CR107] Mondal AK, Lata K, Singh M, Chatterjee S, Chauhan A, Puravankara S, Chattopadhyay K (2022) Cryo-EM elucidates mechanism of action of bacterial pore-forming toxins. Biochim Biophys Acta Biomembr 1864:184013. 10.1016/j.bbamem.2022.184013 35908609 10.1016/j.bbamem.2022.184013

[CR108] Morante K, Bellomio A, Gil-Cartón D et al (2016) Identification of a membrane-bound prepore species clarifies the lytic mechanism of actinoporins. J Biol Chem 291:19210–19219. 10.1074/jbc.M116.734053 27445331 10.1074/jbc.M116.734053 PMC5016661

[CR109] Morante K, Bellomio A, Viguera AR, González-Mañas JM, Tsumoto K, Caaveiro JMM (2019) The isolation of new pore-forming toxins from the sea anemone *Actinia fragacea* provides insights into the mechanisms of actinoporin evolution. Toxins (Basel) 11. 10.3390/toxins11070401 10.3390/toxins11070401 PMC6669745 31295915

[CR110] Mori T, Niki T, Uchida Y et al (2024) A non-toxic equinatoxin-II reveals the dynamics and distribution of sphingomyelin in the cytosolic leaflet of the plasma membrane. Sci Rep 14:16872. 10.1038/s41598-024-67803-2 39043900 10.1038/s41598-024-67803-2 PMC11266560

[CR111] Motone K, Kontogiorgos-Heintz D, Wee J et al (2024) Multi-pass, single-molecule nanopore reading of long protein strands. Nature 633:662–669. 10.1038/s41586-024-07935-7 39261738 10.1038/s41586-024-07935-7 PMC11410661

[CR112] Mukhopadhyay A, Chanakul W, Larpin Y-N et al (2025) Pneumolysin nanopores with 20 nm inner diameter to characterize the size and shape of tau oligomers. bioRxiv. 10.1101/2025.02.07.637128

[CR113] Mutter NL, Volaric J, Szymanski W, Feringa BL, Maglia G (2019) Reversible photocontrolled nanopore assembly. J Am Chem Soc 141:14356–14363. 10.1021/jacs.9b06998 31469268 10.1021/jacs.9b06998 PMC6743218

[CR114] Mutter NL, Huang G, van der Heide NJ, Lucas FLR, Galenkamp NS, Maglia G, Wloka C (2021) Preparation of fragaceatoxin C (FraC) nanopores. Methods Mol Biol 2186:3–10. 10.1007/978-1-0716-0806-7_1 32918725 10.1007/978-1-0716-0806-7_1

[CR115] Negoro S, Shibata N, Tanaka Y et al (2012) Three-dimensional structure of nylon hydrolase and mechanism of nylon-6 hydrolysis. J Biol Chem 287:5079–5090. 10.1074/jbc.M111.321992 22187439 10.1074/jbc.M111.321992 PMC3281642

[CR116] Offenbartl-Stiegert D, Rottensteiner A, Dorey A, Howorka S (2022) A light-triggered synthetic nanopore for controlling molecular transport across biological membranes. Angew Chem Int Ed Engl 61:e202210886. 10.1002/anie.202210886 36318092 10.1002/anie.202210886 PMC10098474

[CR117] Palacios-Ortega J, García-Linares S, Astrand M, Al Sazzad MA, Gavilanes JG, Martínez-del-Pozo A, Slotte JP (2016) Regulation of sticholysin II-induced pore formation by lipid bilayer composition, phase state, and interfacial properties. Langmuir 32:3476–3484. 10.1021/acs.langmuir.6b00082 27003246 10.1021/acs.langmuir.6b00082

[CR118] Palacios-Ortega J, García-Linares S, Rivera-de-Torre E, Gavilanes JG, Martínez-del-Pozo A, Slotte JP (2019) Sticholysin, sphingomyelin, and cholesterol: a closer look at a tripartite interaction. Biophys J 116:2253–2265. 10.1016/j.bpj.2019.05.010 31146924 10.1016/j.bpj.2019.05.010 PMC6588727

[CR119] Palacios-Ortega J, Rivera-de-Torre E, García-Linares S, Gavilanes JG, Martínez-del-Pozo A, Slotte JP (2021) Oligomerization of sticholysins from forster resonance energy transfer. Biochemistry 60:314–323. 10.1021/acs.biochem.0c00840 33445865 10.1021/acs.biochem.0c00840 PMC8023573

[CR120] Palacios-Ortega J, García-Linares S, Rivera-de-Torre E, Heras-Márquez D, Gavilanes JG, Slotte JP, Martínez-del-Pozo A (2021) Structural foundations of sticholysin functionality. Biochimica Et Biophysica Acta (BBA) - Proteins and Proteomics. 10.1016/j.bbapap.2021.140696 34246789 10.1016/j.bbapap.2021.140696

[CR121] Panchal RG, Smart ML, Bowser DN, Williams DA, Petrou S (2002) Pore-forming proteins and their application in biotechnology. Curr Pharm Biotechnol 3:99–115. 10.2174/1389201023378418 12022262 10.2174/1389201023378418

[CR122] Parker MW, Feil SC (2005) Pore-forming protein toxins: from structure to function. Prog Biophys Mol Biol 88:91–142. 10.1016/j.pbiomolbio.2004.01.009 15561302 10.1016/j.pbiomolbio.2004.01.009

[CR123] Pedrera L, Fanani ML, Ros U, Lanio ME, Maggio B, Álvarez C (2014) Sticholysin I-membrane interaction: an interplay between the presence of sphingomyelin and membrane fluidity. Biochim Biophys Acta 1838:1752–1759. 10.1016/j.bbamem.2014.03.011 24680653 10.1016/j.bbamem.2014.03.011

[CR124] Pedrera L, Gomide AB, Sánchez RE et al (2015) The presence of sterols favors sticholysin I-membrane association and pore formation regardless of their ability to form laterally segregated domains. Langmuir 31:9911–9923. 10.1021/acs.langmuir.5b01687 26273899 10.1021/acs.langmuir.5b01687

[CR125] Pettersen EF, Goddard TD, Huang CC, Couch GS, Greenblatt DM, Meng EC, Ferrin TE (2004) UCSF chimera–a visualization system for exploratory research and analysis. J Comput Chem 25:1605–1612. 10.1002/jcc.20084 15264254 10.1002/jcc.20084

[CR126] Purnell RF, Schmidt JJ (2009) Discrimination of single base substitutions in a DNA strand immobilized in a biological nanopore. ACS Nano 3:2533–2538. 10.1021/nn900441x 19694456 10.1021/nn900441x

[CR127] Rivera-de-Torre E, García-Linares S, Alegre-Cebollada J, Lacadena J, Gavilanes JG, Martínez-del-Pozo A (2016) Synergistic action of actinoporin isoforms from the same sea anemone species assembled into functionally active heteropores. J Biol Chem 291:14109–14119. 10.1074/jbc.M115.710491 27129251 10.1074/jbc.M115.710491 PMC4933170

[CR128] Rivera-de-Torre E, Palacios-Ortega J, García-Linares S, Gavilanes JG, Martínez-del-Pozo A (2017) One single salt bridge explains the different cytolytic activities shown by actinoporins sticholysin I and II from the venom of *Stichodactyla helianthus*. Arch Biochem Biophys 636:79–89. 10.1016/j.abb.2017.11.005 29138096 10.1016/j.abb.2017.11.005

[CR129] Rivera-de-Torre E, Palacios-Ortega J, Gavilanes JG, Martínez-del-Pozo A, García-Linares S (2019) Pore-forming proteins from cnidarians and arachnids as potential biotechnological tools. Toxins (Basel) 11. 10.3390/toxins11060370 10.3390/toxins11060370 PMC6628452 31242582

[CR130] Rivera-de-Torre E, Palacios-Ortega J, Garb JE, Slotte JP, Gavilanes JG, Martínez-del-Pozo A (2020) Structural and functional characterization of sticholysin III: a newly discovered actinoporin within the venom of the sea anemone *Stichodactyla helianthus*. Arch Biochem Biophys 689:108435. 10.1016/j.abb.2020.108435 32485153 10.1016/j.abb.2020.108435

[CR131] Robles-Martín A, Amigot-Sánchez R, Fernandez-Lopez L et al (2023) Sub-micro- and nano-sized polyethylene terephthalate deconstruction with engineered protein nanopores. Nat Catal 6:1174–1185. 10.1038/s41929-023-01048-6

[CR132] Robles-Martín A, Roda S, Muñoz-Tafalla R, Guallar V (2024) Behind the scenes of plurizyme designs. Eng 5:91–103. 10.3390/eng5010006

[CR133] Roda S, Fernandez-Lopez L, Benedens M et al (2022) A plurizyme with transaminase and hydrolase activity catalyzes cascade reactions. Angew Chem Int Ed 61. 10.1002/anie.202207344 10.1002/anie.202207344 PMC9540564 35734849

[CR134] Rojko N, Kristan KC, Viero G, Zerovnik E, Maček P, Dalla Serra M, Anderluh G (2013) Membrane damage by an α-helical pore-forming protein, equinatoxin II, proceeds through a succession of ordered steps. J Biol Chem 288:23704–23715. 10.1074/jbc.M113.481572 23803608 10.1074/jbc.M113.481572 PMC3745318

[CR135] Rosenboom JG, Langer R, Traverso G (2022) Bioplastics for a circular economy. Nat Rev Mater 7:117–137. 10.1038/s41578-021-00407-8 35075395 10.1038/s41578-021-00407-8 PMC8771173

[CR136] Ruan Y, Rezelj S, Bedina Zavec A, Anderluh G, Scheuring S (2016) Listeriolysin O membrane damaging activity involves arc formation and lineaction – implication for *Listeria monocytogenes* escape from phagocytic vacuole. PLoS Pathog 12:e1005597. 10.1371/journal.ppat.1005597 27104344 10.1371/journal.ppat.1005597 PMC4841516

[CR137] Sadler JC, Wallace S (2021) Microbial synthesis of vanillin from waste poly(ethylene terephthalate). Green Chem 23:4665–4672. 10.1039/d1gc00931a 34276250 10.1039/d1gc00931a PMC8256426

[CR138] Schön P, García-Saez AJ, Malovrh P, Bacia K, Anderluh G, Schwille P (2008) Equinatoxin II permeabilizing activity depends on the presence of sphingomyelin and lipid phase coexistence. Biophys J 95:691–698. 10.1529/biophysj.108.129981 18390598 10.1529/biophysj.108.129981 PMC2440466

[CR139] Schymanski D, Ossmann BE, Benismail N et al (2021) Analysis of microplastics in drinking water and other clean water samples with micro-Raman and micro-infrared spectroscopy: minimum requirements and best practice guidelines. Anal Bioanal Chem 413:5969–5994. 10.1007/s00216-021-03498-y 34283280 10.1007/s00216-021-03498-y PMC8440246

[CR140] Skocaj M, Bakrač B, Krizaj I, Maček P, Anderluh G, Sepcic K (2012) The sensing of membrane microdomains based on pore-forming toxins. Curr Med Chem 20:491–501. 10.2174/0929867311320040002 10.2174/0929867311320040002 23244522

[CR141] Son HF, Cho IJ, Joo S et al (2019) Rational protein engineering of thermo-stable PETase from for highly efficient PET degradation. ACS Catal 9:3519–3526. 10.1021/acscatal.9b00568

[CR142] Song L, Hobaugh MR, Shustak C, Cheley S, Bayley H, Gouaux JE (1996) Structure of staphylococcal α-hemolysin, a heptameric transmembrane pore. Science 274:1859–1866. 10.1126/science.274.5294.1859 8943190 10.1126/science.274.5294.1859

[CR143] Soskine M, Biesemans A, Moeyaert B, Cheley S, Bayley H, Maglia G (2012) An engineered ClyA nanopore detects folded target proteins by selective external association and pore entry. Nano Lett 12:4895–4900. 10.1021/nl3024438 22849517 10.1021/nl3024438 PMC3440510

[CR144] Stoddart D, Heron AJ, Mikhailova E, Maglia G, Bayley H (2009) Single-nucleotide discrimination in immobilized DNA oligonucleotides with a biological nanopore. Proc Natl Acad Sci U S A 106:7702–7707. 10.1073/pnas.0901054106 19380741 10.1073/pnas.0901054106 PMC2683137

[CR145] Stoddart D, Ayub M, Hofler L et al (2014) Functional truncated membrane pores. Proc Natl Acad Sci U S A 111:2425–2430. 10.1073/pnas.1312976111 24469792 10.1073/pnas.1312976111 PMC3932856

[CR146] Sullivan KP, Werner AZ, Ramirez KJ et al (2022) Mixed plastics waste valorization through tandem chemical oxidation and biological funneling. Science 378:207–211. 10.1126/science.abo4626 36227984 10.1126/science.abo4626

[CR147] Surm JM, Landau M, Columbus-Shenkar YY, Moran Y (2024) Sea anemone membrane attack complex/perforin superfamily demonstrates an evolutionary transitional state between venomous and developmental functions. Mol Biol Evol 41. 10.1093/molbev/msae082 10.1093/molbev/msae082 PMC11090067 38676945

[CR148] Tabata A, Ohkubo Y, Sakakura E, Tomoyasu T, Ohkura K, Nagamune H (2012) Investigation of a bacterial pore-forming chimera toxin for application as a novel drug-delivery system tool. Anticancer Res 32:2323–2329 22641669

[CR149] Takiguchi S, Takeuchi N, Shenshin V et al (2025) Harnessing DNA computing and nanopore decoding for practical applications: from informatics to microRNA-targeting diagnostics. Chem Soc Rev 54:8–32. 10.1039/d3cs00396e 39471098 10.1039/d3cs00396e PMC11521203

[CR150] Tanaka K, Caaveiro JM, Tsumoto K (2015) Bidirectional transformation of a metamorphic protein between the water-soluble and transmembrane native states. Biochemistry 54:6863–6866. 10.1021/acs.biochem.5b01112 26544760 10.1021/acs.biochem.5b01112

[CR151] Tanaka K, Caaveiro JM, Morante K, González-Mañas JM, Tsumoto K (2015) Structural basis for self-assembly of a cytolytic pore lined by protein and lipid. Nat Commun 6:6337. 10.1038/ncomms7337 25716479 10.1038/ncomms7337 PMC4351601

[CR152] Team BD (2023) Blender (version 4.0.0). https://www.blender.org. Accessed 20 Oct 2025

[CR153] Tejuca M, Serra MD, Ferreras M, Lanio ME, Menestrina G (1996) Mechanism of membrane permeabilization by sticholysin I, a cytolysin isolated from the venom of the sea anemone *Stichodactyla helianthus*. Biochemistry 35:14947–14957. 10.1021/bi960787z 8942660 10.1021/bi960787z

[CR154] Tejuca M, Dalla Serra M, Potrich C, Álvarez C, Menestrina G (2001) Sizing the radius of the pore formed in erythrocytes and lipid vesicles by the toxin sticholysin I from the sea anemone *Stichodactyla helianthus*. J Membr Biol 183:125–135. 10.1007/s00232-001-0060-y 11562794 10.1007/s00232-001-0060-y

[CR155] Tournier V, Topham CM, Gilles A et al (2020) An engineered PET depolymerase to break down and recycle plastic bottles. Nature 580:216. 10.1038/s41586-020-2149-4 32269349 10.1038/s41586-020-2149-4

[CR156] Turak O, Gagsteiger A, Upadhyay A et al (2025) A third type of PETase from the marine Halopseudomonas lineage. Protein Sci 34:e70305. 10.1002/pro.70305 40960396 10.1002/pro.70305 PMC12442448

[CR157] Ulhuq FR, Mariano G (2022) Bacterial pore-forming toxins. Microbiology (Reading) 168. 10.1099/mic.0.001154 10.1099/mic.0.001154 PMC9558359 35333704

[CR158] Valle A, Alvarado-Mesen J, Lanio ME, Alvarez C, Barbosa JA, Pazos IF (2015) The multigene families of actinoporins (part I): isoforms and genetic structure. Toxicon 103:176–187. 10.1016/j.toxicon.2015.06.028 26187849 10.1016/j.toxicon.2015.06.028

[CR159] Valle A, Hervis YP, Socas LB et al (2016) The multigene families of actinoporins (part II): strategies for heterologous production in *Escherichia coli*. Toxicon 118:64–81. 10.1016/j.toxicon.2016.03.018 27080349 10.1016/j.toxicon.2016.03.018

[CR160] Van Pee K, Neuhaus A, D'Imprima E, Mills DJ, Kuhlbrandt W, Yildiz O (2017) CryoEM structures of membrane pore and prepore complex reveal cytolytic mechanism of Pneumolysin. Elife 6. 10.7554/eLife.23644 10.7554/eLife.23644 PMC5437283 28323617

[CR161] Varanda W, Finkelstein A (1980) Ion and nonelectrolyte permeability properties of channels formed in planar lipid bilayer membranes by the cytolytic toxin from the sea anemone, *Stoichactis helianthus*. J Membr Biol 55:203–211. 10.1007/BF01869461 6106065 10.1007/BF01869461

[CR162] Versloot RCA, Lucas FLR, Yakovlieva L et al (2022) Quantification of protein glycosylation using nanopores. Nano Lett 22:5357–5364. 10.1021/acs.nanolett.2c01338 35766994 10.1021/acs.nanolett.2c01338 PMC9284675

[CR163] Vidal P, Robles-Martín A, Fernandez-Lopez L et al (2024) Unlocking a key residue in a lipase for efficient polyethylene terephthalate (PET) hydrolysis and influencing depolymerization product profiles. ChemCatChem 16:e202400765. 10.1002/cctc.202400765

[CR164] Vidal P, Gimenez-Dejoz J, Fernandez-Lopez L et al (2025) Computationally guided genome rewiring of *Escherichia coli* and its application for nanopolyethylene terephthalate (PET) biodegradation and upcycling. Trends Biotechnol. 10.1016/j.tibtech.2025.07.008 40817029 10.1016/j.tibtech.2025.07.008

[CR165] Volaric J, van der Heide NJ, Mutter NL et al (2024) Visible light control over the cytolytic activity of a toxic pore-forming protein. ACS Chem Biol 19:451–461. 10.1021/acschembio.3c00640 38318850 10.1021/acschembio.3c00640 PMC10877574

[CR166] Wacklin HP, Bremec BB, Moulin M et al (2016) Neutron reflection study of the interaction of the eukaryotic pore-forming actinoporin equinatoxin II with lipid membranes reveals intermediate states in pore formation. Biochim Biophys Acta. 10.1016/j.bbamem.2015.12.019 26706098 10.1016/j.bbamem.2015.12.019

[CR167] Wang Y, Yap LL, Chua KL, Khoo HE (2008) A multigene family of *Heteractis magnificalysins* (HMgs). Toxicon 51:1374–1382. 10.1016/j.toxicon.2008.03.005 18423794 10.1016/j.toxicon.2008.03.005

[CR168] Wang HY, Li Y, Qin LX et al (2013) Single-molecule DNA detection using a novel SP1 protein nanopore. Chem Commun (Camb) 49:1741–1743. 10.1039/c3cc38939a 23340583 10.1039/c3cc38939a

[CR169] Wang Y, Zhao Y, Bollas A, Wang Y, Au KF (2021) Nanopore sequencing technology, bioinformatics and applications. Nat Biotechnol 39:1348–1365. 10.1038/s41587-021-01108-x 34750572 10.1038/s41587-021-01108-x PMC8988251

[CR170] Wei R, Bornscheuer UT (2023) Designer catalytic nanopores meet PET nanoparticles. Nat Catal 6:1105–1106. 10.1038/s41929-023-01072-6

[CR171] Wei X, Wen J, Wu H, Qu Z, Huang G (2025) Obtaining narrow distributions of single-molecule peptide signals enables sensitive peptide discrimination with α-hemolysin nanopores. J Am Chem Soc 147:9304–9315. 10.1021/jacs.4c15469 40063886 10.1021/jacs.4c15469

[CR172] Wloka C, Mutter NL, Soskine M, Maglia G (2016) α-helical fragaceatoxin C nanopore engineered for double-stranded and single-stranded nucleic acid analysis. Angew Chem Int Ed Engl 55:12494–12498. 10.1002/anie.201606742 27608188 10.1002/anie.201606742

[CR173] Wright SS, Kumari P, Fraile-Agreda V et al (2025) Transplantation of gasdermin pores by extracellular vesicles propagates pyroptosis to bystander cells. Cell 188(280–291):e217. 10.1016/j.cell.2024.11.018 10.1016/j.cell.2024.11.018 PMC12272064 39742811

[CR174] Xia S, Zhang Z, Magupalli VG et al (2021) Gasdermin D pore structure reveals preferential release of mature interleukin-1. Nature 593:607–611. 10.1038/s41586-021-03478-3 33883744 10.1038/s41586-021-03478-3 PMC8588876

[CR175] Yachi R, Uchida Y, Balakrishna BH, Anderluh G, Kobayashi T, Taguchi T, Arai H (2012) Subcellular localization of sphingomyelin revealed by two toxin-based probes in mammalian cells. Genes Cells 17:720–727. 10.1111/j.1365-2443.2012.01621.x 22747662 10.1111/j.1365-2443.2012.01621.x

[CR176] Yamaji M, Chinappi M, Morozzo Della Rocca B, Usui K, Kawano R (2025) Complex and non-sequential current signatures of a beta-hairpin peptide confined in a nanopore. Anal Chem 97:2044–2051. 10.1021/acs.analchem.4c04150 39841857 10.1021/acs.analchem.4c04150 PMC11800182

[CR177] Yan N (2022) Recycling plastic using a hybrid process. Science 378:132–133. 10.1126/science.ade5658 36227992 10.1126/science.ade5658

[CR178] Yang T, Luo J, Nowack B (2021) Characterization of nanoplastics, fibrils, and microplastics released during washing and abrasion of polyester textiles. Environ Sci Technol 55:15873–15881. 10.1021/acs.est.1c04826 34784483 10.1021/acs.est.1c04826

[CR179] Yang L, Pecastaings G, Drummond C, Elezgaray J (2024) Driving DNA nanopore membrane insertion through dipolar coupling. Nano Lett 24:13481–13486. 10.1021/acs.nanolett.4c02302 39432432 10.1021/acs.nanolett.4c02302

[CR180] Ying YL, Hu ZL, Zhang S et al (2022) Nanopore-based technologies beyond DNA sequencing. Nat Nanotechnol 17:1136–1146. 10.1038/s41565-022-01193-2 36163504 10.1038/s41565-022-01193-2

[CR181] York A (2020) Adapting to plastic. Nat Rev Microbiol 18:362–363. 10.1038/s41579-020-0387-y 32440010 10.1038/s41579-020-0387-y

[CR182] Yoshida S, Hiraga K, Takehana T et al (2016) A bacterium that degrades and assimilates poly(ethylene terephthalate). Science 351:1196–1199. 10.1126/science.aad6359 26965627 10.1126/science.aad6359

[CR183] Yu XL, Ni T, Munson G, Zhang PJ, Gilbert RJC (2022) Cryo-EM structures of perforin-2 in isolation and assembled on a membrane suggest a mechanism for pore formation. EMBO J 41. 10.15252/embj.2022111857 10.15252/embj.2022111857 PMC9713709 36245269

[CR184] Zhang F, Zeng MH, Yappert RD et al (2020) Polyethylene upcycling to long-chain alkylaromatics by tandem hydrogenolysis/aromatization. Science 370:437–441. 10.1126/science.abc5441 33093105 10.1126/science.abc5441

[CR185] Zhang M, Chen C, Zhang Y, Geng J (2022) Biological nanopores for sensing applications. Proteins. 10.1002/prot.26308 35092317 10.1002/prot.26308

[CR186] Zhang H, Dierkes RF, Perez-Garcia P et al (2024) The metagenome-derived esterase PET40 is highly promiscuous and hydrolyses polyethylene terephthalate (PET). FEBS J 291:70–91. 10.1111/febs.16924 37549040 10.1111/febs.16924

[CR187] Zhong X, Zeng H, Zhou ZW et al (2023) Structural mechanisms for regulation of GSDMB pore-forming activity. Nature. 10.1038/s41586-023-05872-5 36991125 10.1038/s41586-023-05872-5

